# A Systematic Review of Autoimmunity in 22q11.2 Deletion Syndrome

**DOI:** 10.1017/erm.2026.10031

**Published:** 2026-01-08

**Authors:** Hidayat Y. Ogunsola, Sana Malik, Hannah Rogers, Brad D. Pearce

**Affiliations:** 1 Rollins School of Public Health, Emory University, Atlanta, USA; 2 School of Medicine, Medical Sciences and Nutrition, University of Aberdeen, UK; 3 Library (Head of Information Services), Woodruff Health Sciences Center, Emory University, Atlanta, USA; 4 Epidemiology, Rollins School of Public Health, Emory University, Atlanta, USA

**Keywords:** 22q.11DS, 22q11.2DS, autoimmune, autoimmune thyroid disease, autoimmunity, DiGeorge, DiGeorge syndrome, systematic review

## Abstract

**Background:**

The 22q11.2 Deletion Syndrome (22q11DS) is the most common chromosomal microdeletion disorder, characterised by a heterogeneous clinical spectrum including immunodeficiency, autoimmunity, and neuropsychiatric comorbidities. This systematic review critically appraises current evidence on autoimmunity in 22q11DS, fulfilling the need for an unbiased and comprehensive synthesis of the current literature.

**Methods:**

An extensive search was conducted through PubMed, Web of Science, EMBASE, CINAHL, and the Cochrane Library using Boolean combinations of relevant keywords. Qualitative studies, abstracts, conference proceedings and non-English studies were excluded.

**Results:**

A total of 82 peer-reviewed studies published since 1968 were identified. We identified a total of 40 distinct autoimmune conditions involving multiple organ systems. Haematological disorders were most frequently cited, followed by autoimmune thyroid diseases and systemic autoimmune diseases. Less common conditions included coeliac disease, psoriasis, vitiligo, alopecia areata, Raynaud’s phenomenon, and vasculitis, while 19 diseases appeared only as single-case reports. Neuropsychiatric manifestations were addressed in 24 studies.

**Conclusion:**

Our review confirms that autoimmunity is a complication of 22q11DS and highlights the need for epidemiological studies across organ-systems and inclusion of ethnically diverse populations. There was substantial variation in study designs, underscoring the need for more standardised approaches and larger sample sizes.

## Introduction

The 22q11.2 deletion syndrome (22q11DS) is a recurrent copy number variant disorder that affects approximately 1 in 2,000–6,000 births (Refs 1, 2). Common complications of 22q11DS include conotruncal cardiac defects and subsequent neonatal hypoxia, endocrine dysregulation, including hypocalcemia secondary to parathyroid insufficiency, cleft palate and immune abnormalities (Ref. 3). None of those features occurs invariably in every affected patient, but the factors that determine the particular combination of outcomes in any individual case remain obscure.

In approximately 87% of 22q11DS patients, the hemizygous deletion occurs in a 3 megabases (3 Mb) ‘typically deleted’ region (TDR) containing 46 unambiguous protein-encoding genes (Refs 4, 5). In approximately 10% of patients, there is a nested deletion of approximately 1.5 Mb that uses the same proximal break point but a different distal break point that lays within the ‘typically deleted’ 3 Mb region (Refs 6-8). Because one copy of the affected genes is still present in 22q11DS patients, the disease can be viewed as dysregulation (vs. obliteration) of pathways entailing these genes.

Clinically, patients with 22q11DS have a diminished number and function of T lymphocytes, and can present with a variety of immunological disorders that vary in their features, severity and changes with ageing (Ref. 9). Much of the focus has been on T-cell defects but other immune cells, including B-cells, are also affected (Refs 10, 11).

The immune abnormalities in 22q11DS are often attributed to thymic aplasia or hypoplasia, which occurs due to improper migration of neural crest cells during embryonic development (Ref. 12). Despite a decreased number of T-cells, several autoimmune diseases have been described in the syndrome (Ref. 12). The exact mechanism responsible for autoimmunity in 22q11DS is complex and likely varies among individuals. Possible mechanisms include impaired central negative selection of T cells, reduced number or function of Tregs and altered dendritic cell populations (Refs 12, 13). There is also evidence of increased percentages of inflammatory Th1, Th17 and memory T-helper cells in adults (Ref. 14).

Thymic hormones (e.g., thymosin, thymopoietin and thymulin) play an essential role in regulating T-cell differentiation and maturation and could play a role in the autoimmune aspects of 22q11DS (Ref. 15). These hormones are released into the general circulation and have a range of effects on other immune cells (NK, macrophages) as well as cytokines (Ref. 15). Thus, the deficiency of thymus-derived hormones in 22q11DS could have effects beyond the thymus itself. In autoimmune diseases, such as lupus and autoimmune arthritis, there is a decrement in circulating thymosin α1 levels (Ref. 16), and this hormone has been suggested as a possible therapeutic for autoimmunity (Ref. 15). In 22q11DS, the administration of thymosin has been used with the aim of enhancing T-cell number and function, although it is not currently used to treat the disorder (Ref. 17).

Although the enhanced risk for autoimmunity in 22q11DS is recognised, the accumulating evidence for this is often from case reports and has not been subjected to a systematic review. We considered the heterogeneity of study designs, populations, sample selection, quality and sample sizes in this first systematic review of the subject. Given that this is a multisystem illness, articles are often published in different specialty journals, and this underscores the need to gather this diverse research and clinical reports in a systematic fashion. In the current article, we provide a comprehensive and unbiased summary of the available literature on the presence of autoimmune disease in 22q11DS.

## Methods

### Eligibility criteria

Peer-reviewed and preprint edition studies that described autoimmune manifestations among people with 22q11DS (including DiGeorge, Velocardiofacial, Shprintzen and CATCH 22 Syndromes) and other conditions resulting from the same 22q11 microchromosomal deletion were selected. Autoimmune conditions were derived from the list of autoimmune disease referenced from the National Institutes of Health (NIH) (Ref. 18). Exclusion criteria included patients without 22q11.2 deletion, studies that did not report autoimmune outcomes, qualitative studies, conference proceedings, abstracts, studies published before 1968 and those not written in English.

### Literature search

A comprehensive search for eligible studies was conducted in PubMed, Web of Science, EMBASE, CINAHL and Cochrane (Reviews and Trials) databases and was completed in October 2023 (Table 1). Search terms were divided into those for autoimmune conditions and for 22q11DS, with Boolean operators to search combinations of keywords. Full search strings can be found in Supplementary Table 1.

**Table 1.** Databases and eligible studies on autoimmunity in 22q11DS

Database	Date searched	Results
PubMed	10 April 2023	137
Web of Science	10 April 2023	342
EMBASE	10 May 2023	188
CINAHL	10 May 2023	47
Cochrane (Reviews and Trials)	10 May 2023	270

### Data extraction

**Figure 1.**
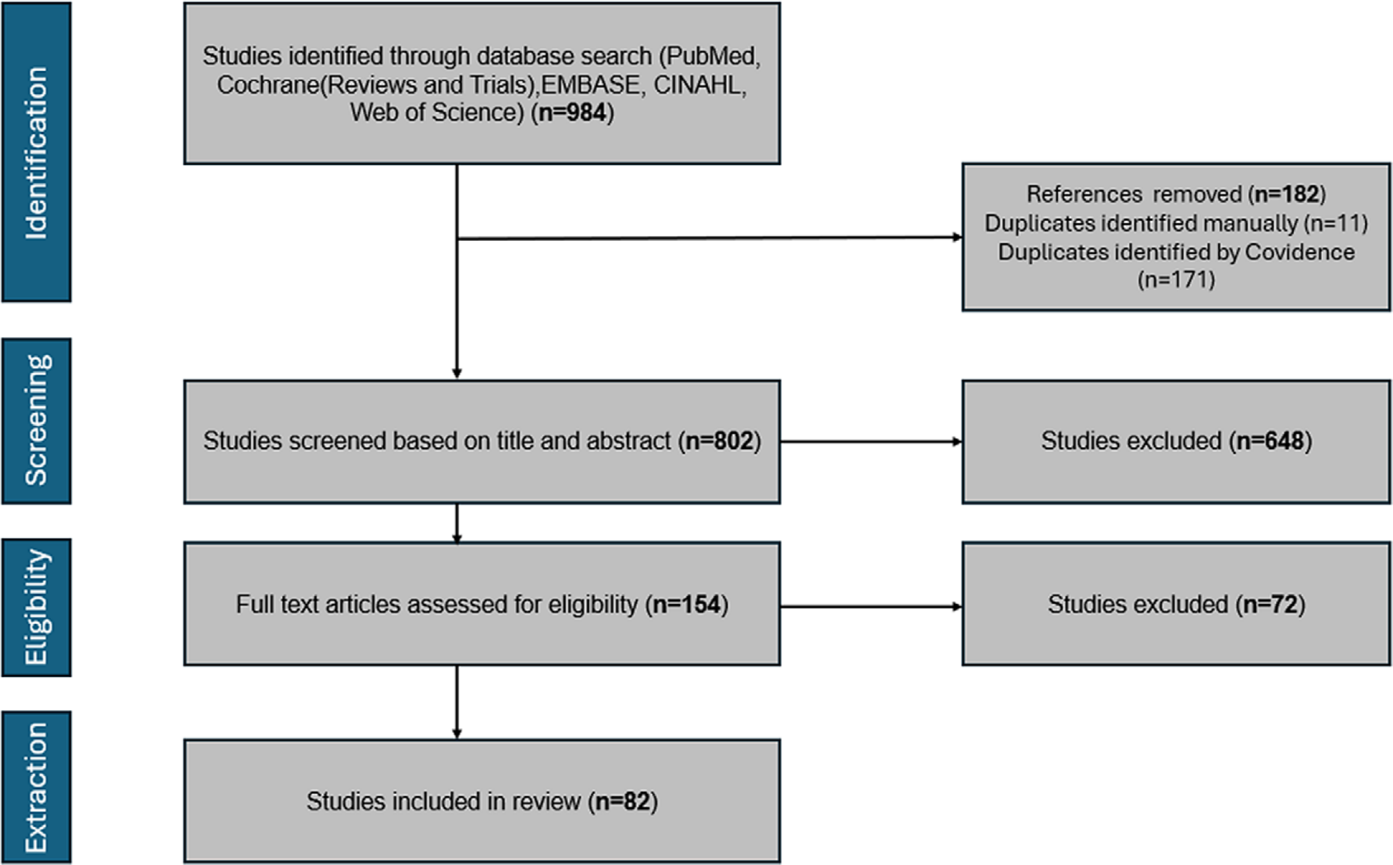
PRISMA flow diagram of systematic review on 22q.11.2 Deletion Syndrome and autoimmunity.

The PRISMA diagram (Figure 1) depicts the selection process. A total of 984 articles were imported into Covidence software, a web tool for managing systematic reviews. There were 182 duplicates removed, and two reviewers conducted independent screening of titles and abstracts using established inclusion and exclusion criteria.

This was followed by independent full-text screening of 154 studies. A third reviewer resolved any conflicts. Full-text screening involved deeper scrutiny of reported autoimmune diseases, details of the study population and study design. Autoimmune diseases corresponded to the list of conditions reported by the NIH (Ref. 18) to ensure standardisation and clinical relevance. Reasons for exclusion were documented at each stage of the screening process.

In the final review, 82 articles were included. Case reports and case series assessing autoimmune features in 22q11DS patients were included. Data were extracted systematically by defined study, participant and autoimmune diagnostic characteristics. Study characteristics extracted include title of the paper, lead author, year of publication, journal name, country in which study was conducted, specific geographic location, and aim of study. Data on methods included study design, funding sources, author conflict of interest, number of participants and inclusion and exclusion criteria. We collected participant data, including gender, race and/or ethnicity, age (reported the median age; otherwise, reported the mean age) and recruitment method. The presence of neuropsychiatry conditions was also documented. Autoimmune diseases were tabulated among the study population. To further confirm and analyse the reports of autoimmune statuses, information on the type and presence of autoantibodies was collected.

To evaluate transparency and risk of bias, quality assessment was conducted in Covidence using the Newcastle–Ottawa Scale (NOS) (Ref. 19) and an adapted NOS for cross-sectional studies (Ref. 20). For the studies, selection was analysed through the representativeness of the 22q11DS group (exposed), the selection of the comparison or control group, the definition of 22q11DS and any demonstration that the outcome was not present at baseline. Comparability on the basis of design, assessment of outcome/exposure, follow-up duration and adequacy of follow-up (cohort studies) and nonresponse rate for both groups (case–control) was also included to assess quality.

## Results

There was a heterogeneous mix of study designs. Details of the 82 studies included in our review are in Tables 2 and 3. There were 34 studies that were either case–control, cohort or cross-sectional studies, most of which had an approximately equal male-to-female ratio (Table 2). Furthermore, 15 studies were retrospective, 10 studies were prospective, 2 studies were both prospective and retrospective, 5 studies were cross-sectional, 1 study was a mixed study (cross-sectional and cohort) and 1 study was not clear (Ref. 21). Cohort studies reporting a percent affected typically used incidence proportion. The majority of the studies were case reports or case series (*n* = 48), with 58.3% of the case reports being female participants (Table 3). Twenty one studies were conducted in North America (18 in the United States, 2 in Canada, and 1 in Mexico), 38 in Europe (7 in UK, 2 in Belgium, 2 in the Czech Republic, 2 in Finland, 4 in France, 1 in Germany, 1 in Greece, 14 in Italy, 1 in the Netherlands, 2 in Norway, 1 in Spain, and 1 in Sweden), 22 in Asia (1 in China, 1 in Iran, 3 in Israel, 8 in Japan, 1 in Korea, 1 in Kuwait, 1 in Qatar, 1 in South Korea, 2 in Taiwan, and 3 in Turkey) and 1 in South America (Brazil) (Tables 2 and 3). Notably, no studies have been reported from the African continent.

**Table 2.** Cohort, case–control and cross-sectional studies on autoimmunity in 22q11.2 deletion syndrome

Author	Country	Article	Age	Participants	% (*n* ^a^) Autoimmune disease	Design/year of study	Autoantibodies/neuropsychiatric manifestations
Bassett et al., 2005 (Ref. 33)	Canada	Clinical features of 78 adults with 22q11 deletion syndrome	Mean age 31.5 years	78	6.4% autoimmunity 5 idiopathic thrombocytopenic purpura	Cross-sectional –	No autoantibody reported Psychiatric disorders 8, schizophrenia 31
Björk et al., 2012 (Ref. 45)	Sweden	Antibody deficiency in adults with 22q11.2 deletion syndrome	Median age 24.5 years Range 18–44 years	26	15.4% autoimmunity 4 ITP, 3 autoimmune haemolytic anaemia, 1 juvenile idiopathic arthritis	Cohort Retrospective & Prospective 1997–2007	No autoantibody or NP/ND reported
Cancrini et al., 2014 (Ref. 46)	Italy	Clinical features and follow-up in patients with 22q11.2 deletion syndrome	Median age 4 months Range 0–36 years 10 months	228	23% autoimmunity 12 thrombocytopenia, 2 autoimmune haemolytic anaemia, 16 thyroiditis, 5 arthritides, 1 hepatitides, 10 other (not specified)	Cohort Retrospective & Prospective 2006–2012	No autoantibody reported Speech and language delay 70%, psychomotor developmental delay 53%, learning difficulties 65%, behavioural abnormalities 11%, psychiatric disorders 7%, other 1%
Choi et al., 2005 (Ref. 47)	Korea	Endocrine manifestations of chromosome 22q11.2 microdeletion syndrome	Mean age 1.9 years Range 15 days to 12 years	61	3.3% autoimmunity 1 (1.6%) Graves’ disease, 1 (1.6%) Hashimoto’s thyroiditis	Cohort Prospective –	TSH receptor antibodies, thyroid autoantibody: antithyroglobulin antibody and microsome antibody No NP/ND reported
Crowley et al., 2022 (Ref. 48)	United States	Distinct immune trajectories in patients with chromosome 22q11.2 deletion syndrome and immune-mediated diseases	>18 years	1,533	2.0% autoimmunity 18 autoimmune thyroiditis (Graves’ disease or Hashimoto’s thyroiditis), 5 juvenile idiopathic arthritis, 7 immune thrombocytopenia, 2 autoimmune haemolytic anaemia, 1 nephritis	Cohort Retrospective 1992–2020	Positive autoantibody ADHD 52%, autism 19%, seizures 15%, psychotic disorder 15%
Deshpande et al., 2021 (Ref. 49)	United States	Relationship between severity of T cell lymphopenia and immune dysregulation in patients with DiGeorge syndrome (22q11.2 deletions and/or related TBX1 mutations)	Median age 4.6 years Range 0–45.6 years	415	4.1% autoimmunity 6 immune cytopenia (ITP or HA), 5 immune thrombocytopenia (ITP), 4 haemolytic anaemia (HA), 4 hypothyroidism, 4 hyperthyroidism, 2 psoriasis, 2 rheumatologic conditions (JIA, systemic sclerosis), 1 diabetes mellitus, 1 vitiligo, 1 alopecia	Cohort Retrospective –	No autoantibody or NP/ND reported
DiCesare et al., 2015 (Ref. 50)	Italy	Autoimmunity and regulatory T cells in 22q11.2 deletion syndrome patients	Median 5 years Range 0–17 years	50 + 22 control	18% autoimmunity 3 juvenile idiopathic arthritis, 1 thyroiditis, 3 autoimmune thrombocytopenia, 2 psoriasis, 1 Coeliac disease, 1 autoimmune hepatitis 2 AHA	Cohort Prospective –	No autoantibody or NP/ND reported
Digilio et al., 2003 (Ref. 51)	Italy	Screening for celiac disease in patients with deletion 22q11.2 (DiGeorge/velo-cardio-facial syndrome)	>18 months	48	2.1% autoimmunity 1 Coeliac disease	Cohort Prospective 1997–2001	No autoantibody or NP/ND reported
Gennery et al., 2002 (Ref. 10)	UK	Antibody deficiency and autoimmunity in 22q11.2 deletion syndrome	Range 0.67–22 years	32	33% autoimmunity 3 Raynaud’s phenomenon, 1 Evans syndrome, 1 pauciarticular arthritis (now called juvenile idiopathic arthritis), 1 thrombocytopenia (ITP), 1 vasculitis, 1 urticaria	Cohort Prospective 1992–2001	Rheumatoid factor (RhF), ANA, AECA antibodies, antiplatelet antibody, GPC, thyroid microsomal, antiplatelet antibody, cardiolipin, transient SM antibody No NP/ND reported
Giardino et al., 2014 (Ref. 52)	Italy	Gastrointestinal involvement in patients affected with 22q11.2 deletion syndrome	Range 0.6–20.7 years	26	19.2% autoimmunity 1 psoriatic arthritis, 3 Coeliac disease, 1 autoimmune thyroiditis	Cohort Retrospective –	Anti-transglutaminase IgA, anti-gliadin antibodies IgG, ANA, anti-double stranded DNA (anti-dsDNA), extractable nuclear antibodies (ENA), anti-thyroglobulin (anti-Tg), anti-thyroid peroxidase (anti-TPO), anti-gliadin IgA (AGA IgA), anti-gliadin IgG (AGA IgG) No NP/ND reported
Giardino et al., 2019 (Ref. 27)	UK	Clinical and immunological features in a cohort of patients with partial DiGeorge syndrome followed at a single center	Range 0.23–30.4 years	467	7.84% autoimmunity 9 autoimmune cytopenia (5 neutropenia, 4 ITP, 2 AIHA), 7 autoimmune thyroid disease, 6 juvenile idiopathic arthritis, 4 alopecia areata, 3 IBD, 3 Raynaud phenomenon, 3 psoriasis, 2 vitiligo, 1DM, 1SLE/juvenile dermatomyositis, 1 unclassified immune mediated inflammatory lung diseases	Cohort Retrospective 2004–2016	No autoantibody or NP/ND reported
Grinde et al., 2020 (Ref. 53)	Norway	Complement activation in 22q11.2 deletion syndrome	Range 3.5–15 years	69 +56 HC	8.7% autoimmunity 2 Autoimmune type hypothyroidism, 1 diabetes mellitus-type 1, 1 pernicious anaemia and atrophic gastritis, 1 Sjogren’s syndrome, 1 sarcoidosis	Cross sectional 2011–2015	Parietal-cell and intrinsic factor autoantibodies ND: ADHD, ADD, ASD, intellectual disability NP: anxiety, major depressive, psychosis; learning disabilities, developmental delay
Jawad et al., 2001 (Ref. 54)	United States	Immunologic features of chromosome 22q11.2 deletion syndrome (DiGeorge syndrome/velocardiofacial syndrome)	Range 0–9 years	281	8.7% autoimmunity 4 JRA, 8 idiopathic thrombocytopenia purpura, 1 autoimmune haemolytic anaemia, 1 vitiligo, 1 psoriasis, 1 IBD, 1 adult rheumatoid arthritis	Cross-sectional and cohort (mixed-design); included healthy control group –	No autoantibody or NP/ND reported
Klocperk et al., 2018 (Ref. 21)	Czech Republic	Follicular helper T cells in DiGeorge syndrome	Mean 7.6 years Range 0.5–21 years	17	29.4% autoimmunity 5 autoimmune thrombocytopenia	Cohort study NC	No autoantibody or NP/ND reported
Kotcher et al., 2022 (Ref. 55)	United States	Gastrointestinal features of 22q11.2 deletion syndrome include chronic motility problems from childhood to adulthood	Range 17–59 years	206	1.5% autoimmunity 1 microscopic colitis	Cohort Retrospective –	No autoantibodies reported Neurodevelopmental disorder, seizure disorder, depressive disorder, anxiety disorder, psychotic disorder, psychosis
Legitimo et al., 2020 (Ref. 56)	Italy	Vitamin D status and the immune assessment in 22q11.2 deletion syndrome	Range 2–18 years	17 +17 HC	47% autoimmunity 1 alopecia, 1 psoriasis, 2 thrombocytopenia, 5 thyroiditis	Cohort Prospective –	No autoantibody or NP/ND reported
Lima et al., 2011 (Ref. 57)	Norway	Hypoparathyroidism and autoimmunity in the 22q11.2 deletion syndrome	Median age 9 years Range 1–54 years	60	10% autoimmunity 1 Raynaud’s phenomenon, 3 hypothyroidism, 1 pernicious anaemia, 1 atrophic gastritis, 2 Coeliac disease, 1 Idiopathic thrombocytopenic purpura, 1 psoriatic arthritis	Cohort study Prospective 1993–2006	Tyrosine Hydroxylase, anti-smooth muscle (ASM), thyroid peroxidase (TPO), thyroglobulin (TG), ANA, tissue transglutaminase (tTG), intrinsic factor antibodies, parietal-cell antibodies No NP/ND reported
Lin et al., 2022 (Ref. 58)	Taiwan	Endocrine and growth disorders in Taiwanese children with 22q11.2	Mean age 2.3 ± 3.2 years Range 1 day to 17 years and 11 months	138	0.7% autoimmunity 1 Graves’ disease	Cohort Retrospective 1994–2020	Anti-thyroperoxidase antibody, anti-thyroglobulin antibody No NP/ND reported
Mahe et al., 2019 (Ref. 59)	France	Risk factors of clinical dysimmune manifestations in a cohort of 86 children with 22q11.2 deletion syndrome: a retrospective study in France	<18 years	86	13% autoimmunity 4 autoimmune thyroiditis, 2 Idiopathic juvenile arthritis, 2 ITP, 1 diabetes, 1 ulcerative colitis, 1 autoimmune neutropenia	Cohort Retrospective 1992–2014	No autoantibody or NP/ND reported
McLean-Tooke et al., 2008 (Ref. 60)	UK	Immunologic defects in 22q11.2 deletion syndrome	Not specified	27 +54 HC	15% autoimmunity	Cross-sectional (include healthy control)	Positive autoantibodies (not specified) No NP/ND reported
Montin et al., 2019 (Ref. 31)	Italy	Immunophenotype anomalies predict the development of autoimmune cytopenia in 22q11.2 deletion syndrome	Not specified	23 +45 control	21 ITP, 7 AIHA, 1 psoriasis, 2 juvenile idiopathic arthritis/rheumatoid arthritis	Case control Retrospective & Prospective 2006–2018	No autoantibody or NP/ND reported
Nain et al., 2019 (Ref. 61)	Turkey	Immune system defects in DiGeorge syndrome and association with clinical course	Median age 1 month Range 1–18 years	18	27.7% autoimmunity 1 psoriasis, 2 idiopathic thrombocytopenic purpura, 1 autoimmune haemolytic anaemia, 1 juvenile idiopathic arthritis	Cohort Prospective –	No autoantibody or NP/ND reported
Nissan et al., 2021 (Ref. 23)	Israel	Clinical features in a large cohort of patients with 22q11.2 deletion syndrome	Median age 2 years Range 0.0–36.5 years	98	10% autoimmunity 9 autoimmune diseases	Cohort Retrospective –	No autoantibody or NP/ND reported
Ongen et al., 2023 (Ref. 26)	Turkey	An endocrinological perspective on 22q11.2 deletion syndrome: a single-center experience	Median age 45 days Range 1 day to 13 years	17	11.7% autoantibodies 2 autoantibodies	Cohort Retrospective –	Thyroid autoantibodies, anti-thyroid peroxidase (TPO), anti-thyroglobulin (TG) No NP/ND reported
Ozen et al., 2021 (Ref. 62)	Turkey	22q11.2 deletion syndrome: 20 years of experience from two pediatric immunology units and review of clues for diagnosis and disease management	Mean age 29.8 ± 39.2 months	33	12.1% autoimmunity 2 autoimmune pancytopenia, 1 autoimmune thyroiditis, 1 autoimmune thrombocytopenia, 1 autoimmune arthritis	Cohort Retrospective 1998–2019	No autoantibody or NP/ND reported
Ricci et al., 2018 (Ref. 63)	Italy	Reduced frequency of peripheral CD4+CD45RA+CD31+ cells and autoimmunity phenomena in patients affected by Del22q11 syndrome	Mean age 13.3 ± 6.2 years	44 +45 HC	22.7% autoimmunity 1 Coeliac disease, 6 hypothyroidism, 1 Evans syndrome, 1 alopecia, 1 vitiligo	Cohort Prospective 2015–2017	Positive for antinuclear autoantibodies, thyroid autoantibodies No NP/ND reported
Ricci et al., 2022 (Ref. 28)	Italy	Characterization of autoimmune thyroid disease in a cohort of 73 pediatric patients affected by 22q11.2 deletion syndrome: longitudinal single-centre study	Range 0–18 years	73	21.9% autoimmunity 15 Hashimoto’s thyroiditis, 1 Grave’s disease	Cohort Prospective 1985–2022	Anti-thyroid peroxidase antibodies (TPOAb), thyroglobulin antibodies (TGAb), TSH receptor antibodies (TRAb) No NP/ND reported
Sedivá et al., 2005 (Ref. 25)	Czech Republic	Early development of immunity in DiGeorge syndrome	Range 4 days–19 years	34	2 autoantibodies	Cohort Prospective 1997	Positive antithyroglobulin antibodies, transient positivity of antinuclear antibodies No NP/ND reported
Stagi et al., 2010 (Ref. 64)	Italy	Thyroid function and morphology in subjects with microdeletion of chromosome 22q11 (del(22)(q11))	Median age 9.7 years Range 1.5–43.9 years	30 + 277 HC	3.3% autoimmunity 1 autoimmune thyroid disease	Cross-sectional + control that included a healthy control group 2005–2008	3 thyroid autoantibodies No NP/ND reported
Sullivan et al., 1997 (Ref. 38)	United States	Juvenile rheumatoid arthritis-like polyarthritis in chromosome 22q11.2 deletion syndrome (DiGeorge anomalad/velocardiofacial syndrome/conotruncal anomaly face syndrome)	Not specified	80	3.8% autoimmunity 3 JRA-like polyarthritis	Cross-sectional –	No autoantibody or NP/ND reported
Tison et al., 2011 (Ref. 65)	United States	Autoimmunity in a cohort of 130 pediatric patients with partial DiGeorge syndrome	Median age 7.5 years Range 5 months to 23 years	130	8.5% autoimmunity 3 autoimmune hypothyroidism, 1 monoarticular arthritis, 1 vitiligo, 1 juvenile idiopathic arthritis, 1 psoriasis, 1 autoimmune neutropenia	Cohort Retrospective 1987–2011	Anti-thyroglobulin and peroxidase anti-thyroid antibodies, positive antinuclear antibody, antineutrophil antibodies No NP/ND reported
Wahrmann et al., 2023 (Ref. 24)	Finland	Childhood manifestations of 22q11.2 deletion syndrome: A Finnish nationwide register-based cohort study	Median age <1 year	1,078	29.6% autoimmune diseases	Cohort Retrospective 2005–2018	No autoantibodies reported 93.2% neuropsychiatric conditions
Yu et al., 2022 (Ref. 66)	Taiwan	Clinical and immunological defects and outcomes in patients with chromosome 22q11.2 deletion syndrome	Median age 1.78 months Range 0–18.6 years	87	12.6% autoimmunity 9 autoimmune thrombocytopenia, 1 autoimmune pancytopenia, 1 Graves’ disease	Cohort Retrospective 2000–2021	No autoantibody or NP/ND reported
Zama et al., 2021 (Ref. 67)	Italy	Immune cytopenias as a continuum in inborn errors of immunity: An indepth clinical and immunological exploration	0–18 years	47 (only 1 22q participant)	2.1% autoimmunity 1 ITP, AIHA, AIN	Cohort Retrospective 2000–2019	No autoantibody or NP/ND reported

ADD: attention-deficit disorder; ADHD: attention-deficit/hyperactive disorder; AECA: antiendothelial antibody; AIHA: autoimmune haemolytic anaemia; AIN: autoimmune neutropenia; ANA: antinuclear antibody; ASD: autism spectrum disorder; GPC: gastric parietal cell; HC: healthy control; IBD: inflammatory bowel disease; IgA: immunoglobulin A; IgG: immunoglobulin G; ITP: immune thrombocytopenic purpura; JRA: juvenile rheumatoid arthritis; nc: not clear; ND: neurodevelopmental; NP: neuropsychiatric; RA: rheumatoid arthritis; RhF: Rheumatoid factor; SM: smooth muscle.

a *n* is the number in the study with each following autoimmune condition.

**Table 3.** Case reports and case series on autoimmunity in 22q11.2 deletion syndrome

Author	Country	Article	(n)Participants	(n) Autoimmune disease	Autoantibodies
Akar et al., 2007 (Ref. 68)	Kuwait	Chromosome 22q11.2 deletion presenting with immune-mediated cytopenias, macrothrombocytopenia and platelet dysfunction	1	1 immune-mediated thrombocytopenia	ANA, platelet specific antibodies
Arkush et al., 2019 (Ref. 69)	Israel	Glomerulonephritis and nephrotic syndrome in a child with DiGeorge syndrome	1	1 hypocomplementemic glomerulonephritis with nephrotic syndrome	–
Brown et al., 2004 (Ref. 70)	UK	Graves’ disease in DiGeorge syndrome: patient report with a review of endocrine autoimmunity associated with 22q11.2 deletion	1	1 Graves’ disease	Thyroid microsomal, peroxidase, thyroglobulin antibodies, TSH receptor binding antibodies, gastric parietal cell antibodies (no proof of pernicious anaemia)
Bruno et al., 2002 (Ref. 71)	France	Auto-immune pancytopenia in a child with DiGeorge syndrome	1	1 autoimmune pancytopenia	Coombs positive red cells for IgG, platelet-associated antibodies (IgG and IgM) and anti-neutrophil antibodies
Chang et al., 2006 (Ref. 72)	United States	Type III mixed cryoglobulinemia and antiphospholipid syndrome in a patient with partial DiGeorge syndrome	1	1 immune thrombocytopenia, antiphospholipid syndrome, 1 diffuse proliferative glomerulonephritis	Anti-platelet IgM, cardiolipin IgM antibodies, and elevated beta 2-glycoprotein I IgM antibodies
Choi et al., 2021 (Ref. 73)	Japan	Case report: Meniere’s disease-like symptoms in 22q11.2 deletion syndrome	1	1 Meniere’s disease	–
Ciano-Petersen et al., 2021 (Ref. 74)	Spain	Case report: Autoimmune psychosis in chromosome 22q11.2 deletion syndrome	1	1 autoimmune psychosis	–
Colarusso et al., 2010 (Ref. 75)	Italy	Evans syndrome and antibody deficiency: an atypical presentation of chromosome 22q11.2 deletion syndrome	1	1 immune thrombocytopenia, 1 autoimmune haemolytic anaemia, Evans syndrome	Positive direct Coombs test
Comont et al., 2021 (Ref. 76)	France	A complex infectious, inflammatory, and autoimmune phenotype reveals 22q11.2 deletion syndrome in an adult	1	1 immune thrombocytopenia, pyoderma gangrenosum	–
Conti et al., 2022 (Ref. 77)	Italy	Refractory immune thrombocytopenia successfully treated with bortezomib in a child with 22q11.2 deletion syndrome, complicated by Evans syndrome and hypogammaglobulinemia	1	1 immune thrombocytopenia, 1 Evans syndrome	–
Damlaj et al., 2013 (Ref. 78)	Canada	A case of refractory autoimmune hemolytic anemia in a patient with DiGeorge syndrome treated successfully with plasma exchange	1	1 autoimmune haemolytic anaemia, 1 immune thrombocytopenia purpura	Positive direct Coombs test
Davies et al., 2001 (Ref. 79)	UK	Juvenile idiopathic polyarticular arthritis and IgA deficiency in the 22q11 deletion syndrome	5	5 juvenile idiopathic polyarticular arthritis	Rheumatoid factor positive, ANA
Davies et al., 2003 (Ref. 80)	UK	Autoimmune cytopenias in the 22q11.2 deletion syndrome	2	1 autoimmune haemolytic anaemia, 1 Evans syndrome, 1 immune thrombocytopenia	Positive Direct Antiglobulin Test (DAT)
DePiero et al., 1997 (Ref. 81)	United States	Recurrent immune cytopenias in two patients with DiGeorge/velocardiofacial syndrome	2	2 autoimmune haemolytic anaemia, 2 immune thrombocytopenia	Positive antiglobulin test
Elder et al., 2001 (Ref. 82)	United States	Type I diabetes mellitus in a patient with chromosome 22q11.2 deletion syndrome	1	1 type I diabetes	Insulin antibodies
Gelfand et al., 2007 (Ref. 83)	United States	Hypocalcemia as a presenting feature of celiac disease in a patient with DiGeorge syndrome	1	1 coeliac disease	Coeliac disease antibodies
Gottlieb et al., 2010 (Ref. 84)	United States	Uveitis in DiGeorge syndrome: a case of autoimmune ocular inflammation in a patient with deletion 22q11.2	1	1 autoimmune-mediated bilateral granulomatous uveitis	–
Gu et al., 2023 (Ref. 85)	China	Case report: effectiveness of sirolimus in treating partial DiGeorge syndrome with autoimmune lymphoproliferative syndrome (ALPS)-like features	1	1 autoimmune cytopenias (haemolytic anaemia, thrombocytopenia and neutropenia)	–
Hernandez-Nieto et al., 2011 (Ref. 86)	Mexico	Autoimmune thrombocytopenic purpura in partial DiGeorge syndrome: case presentation	1	1 autoimmune thrombocytopenia	Anticardiolipin antibodies, antinuclear antibodies
Hiroshima et al., 2022 (Ref. 87)	Japan	Two cases of 22q11.2 deletion syndrome with decreased serum calcium during recovery following thyrotoxicosis	2	1 Hashimoto’s thyroiditis, 1 Graves’ disease	Anti-TSH receptor antibody
Iijima et al., 2021 (Ref. 88)	Japan	Symptomatic hypocalcemia after treatment for hyperthyroidism in a woman with chromosome 22q11.2 deletion syndrome complicated by Graves’ disease: longitudinal changes in the number of subsets of CD4 and CD8 lymphocytes after thyroidectomy	1	1 Graves’ disease	Thyroid stimulating hormone receptor antibody
Kawame et al., 2001 (Ref. 89)	Japan	Graves’ disease in patients with 22q11.2 deletion	5	5 Graves’ disease	TRAb: thyroid-stimulating hormone receptor antibody, anti-thyroid antibodies such as anti-thyroglobulin and anti-microsomal antibodies
Kawamura et al., 2000 (Ref. 90)	Japan	DiGeorge syndrome with Graves’ disease: a case report	1	1 Graves’ disease	–
Kratz et al., 2003 (Ref. 91)	Germany	Evans syndrome in a patient with chromosome 22q11.2 deletion syndrome: a case report	1	1 autoimmune haemolytic anaemia, immune thrombocytopenia, autoimmune neutropenia, Evans syndrome	Antiglobulin antibodies, anti-platelet antibodies to Ib/IX and IIb/IIIa, granulocyte-specific antibodies
Levy et al., 1997 (Ref. 92)	France	Idiopathic thrombocytopenic purpura in two mothers of children with DiGeorge sequence: a new component manifestation of deletion 22q11?	4	2 ITP	–
Lozano -Chinga et al., 2022 (Ref. 93)	United States	Lymphoma in partial DiGeorge syndrome: report of 2 cases	2	1 autoimmune hepatitis, 1 autoimmune neutropenia, 2 ITP	–
Nakada et al., 2013 (Ref. 94)	Japan	An adult case of 22q11.2 deletion syndrome diagnosed in a 36-year-old woman with hypocalcemia caused by hypoparathyroidism and Hashimoto’s thyroiditis	1	1 Hashimoto’s thyroiditis	Antithyroglobulin antibodies
Nucera et al., 2006 (Ref. 95)	Italy	Antiphospholipid antibodies syndrome associated with hyperhomocysteinemia related to MTHFR gene C677T and A1298C heterozygous mutations in a young man with idiopathic hypoparathyroidism (DiGeorge syndrome)	1	1 antiphospholipid antibodies syndrome	Anti–2-glycoprotein (anti–2-GPI), anti-prothrombin (anti-PT), antiphospholipid antibodies (aPLs)
Okiyama et al., 2005 (Ref. 96)	UK	Juvenile dermatomyositis in association with 22q11.2 deletion syndrome	1	1 dermatomyositis	Antithyroid receptor antibody, antinuclear antibody
Oliveras-Cordero et al., 2020 (Ref. 97)	Puerto Rico	Recurrent Evans syndrome in a patient with 22q11.2 deletion syndrome: an uncommon hematological presentation	1	1 immune thrombocytopenia (ITP), 1 autoimmune haemolytic anaemia (AIHA), 1 Evans syndrome	Anti-small-e antibodies
Pelkonen et al., 2002 (Ref. 98)	Finland	Chronic arthritis associated with chromosome deletion 22q11.2 syndrome	3	3 juvenile idiopathic arthritis	–
Pereira et al., 2003 (Ref. 99)	Netherlands	Successful carnitine therapy for Raynaud’s phenomenon in velo-cardio-facial syndrome	2	2 Raynaud’s phenomenon	–
Pinchas-Hamiel et al., 1994 (Ref. 100)	Israel	Immune hemolytic anemia, thrombocytopenia and liver disease in a patient with DiGeorge syndrome	1	1 immune haemolytic anaemia, 1 thrombocytopenia	Platelet-associated antibodies, smooth muscle antibodies
Pong et al., 1985 (Ref. 101)	United States	DiGeorge syndrome: long-term survival complicated by Graves’ disease	1	1 Graves’ disease	Thyroid microsomal antibodies
Pongpruttipan et al., 2012 (Ref. 102)	United States	Pulmonary extranodal marginal zone lymphoma of mucosa-associated lymphoid tissue associated with granulomatous inflammation in a child with chromosome 22q11.2 deletion syndrome (DiGeorge syndrome)	1	1 Evans syndrome, 1 Lichen planus	–
Rasmussen et al., 1996 (Ref. 103)	United States	Juvenile rheumatoid arthritis in velo-cardio-facial syndrome: coincidence or unusual complication?	2	2 JRA	ANA, rheumatoid factor
Sakamoto et al., 2004 (Ref. 104)	Japan	Refractory autoimmune hemolytic anemia in a patient with chromosome 22q11.2 deletion syndrome	1	1 autoimmune haemolytic anaemia	Direct Coombs test positive
Salehzadeh et al., 2014 (Ref. 105)	Iran	Association of juvenile idiopathic arthritis and DiGeorge syndrome: a case report	1	2 oligoarthritic juvenile idiopathic arthritis	–
Segni et al., 2002 (Ref. 106)	United States	Autoimmune hyperthyroidism in two adolescents with DiGeorge/velocardiofacial syndrome (22q11 deletion)	2	2 autoimmune hyperthyroidism	Anti-peroxidase (TPO) antibodies
Sestan et al., 2020 (Ref. 107)	United States	Adverse effects of TNF inhibitor in a patient with DiGeorge syndrome and juvenile idiopathic arthritis	1	1 juvenile idiopathic arthritis	–
Soares et al., 2020 (Ref. 108)	Brazil	Lymphoproliferative disorder with polyautoimmunity and hypogammaglobulinemia: an unusual presentation of 22q11.2 deletion syndrome	1	1 thrombotic thrombocytopenic purpura, 1 granulomatous lymphocytic interstitial lung disease/lymphoproliferative disorder	Antiglobulin antibodies
Soldatou et al., 2013 (Ref. 109)	Greece	Transient effect of anti-CD20 therapy in a child with 22q11.2 deletion syndrome and severe steroid refractory cytopenias: a case report	1	1 autoimmune haemolytic anaemia, autoimmune cytopenia, 1 autoimmune pancytopenia, 1 Evans syndrome/immune thrombocytopenia	Direct antiglobulin positive
Ueda et al., 2017 (Ref. 110)	Japan	Graves’ disease in pediatric and elderly patients with 22q11.2 deletion syndrome	2	2 Graves’ disease	Anti-TSH receptor antibody, anti-thyroid peroxidase antibodies (TPOAb), thyroglobulin antibodies (TGAb), anti-TSH receptor antibodies (TRAb)
Valenzise et al., 2014 (Ref. 111)	Italy	Post vaccine acute disseminated encephalomyelitis as the first manifestation of chromosome 22q11.2 deletion syndrome in a 15-month-old baby: a case report	1	1 acute disseminated encephalomyelitis autoimmune	–
Vergaelen et al., 2015 (Ref. 112)	Belgium	3 generation pedigree with paternal transmission of the 22q11.2 deletion syndrome: Intrafamilial phenotypic variability	4	1 psoriatic arthritis	–
Verloes et al., 1998 (Ref. 113)	Belgium	Juvenile rheumatoid arthritis and del (22ql 1) syndrome: a non-random association	3	3 JRA	–
Yi et al., 2023 (Ref. 114)	United States	Ustekinumab for psoriasis and psoriatic arthritis in adolescents with 22q11.2 deletion syndrome	2	1 psoriasis, 1 psoriatic juvenile idiopathic arthritis, plaque psoriasis	–
Yousaf et al., 2021 (Ref. 115)	Qatar	Autoimmune hemolytic anemia associated with vitamin B12 deficiency and viral illness in DiGeorge syndrome. Case report and literature review	1	1 autoimmune haemolytic anaemia (AIHA)	Warm IgG antibody-induced autoimmune haemolytic anaemia (AIHA)

AIHA: autoimmune haemolytic anaemia; ANA: antinuclear antibody; IgA: immunoglobuIin A; IgG: immunoglobulin G; IgM: immunoglobulin M; ITP: immune thrombocytopenic purpura; JRA, juvenile rheumatoid arthritis; TSH: thyroid-stimulating hormone.

Regarding racial/ethnic distribution, 18 studies provided race information, out of which approximately 80% of the participants were white. Only 24 studies reported neuropsychiatric or neurodevelopmental conditions, including autoimmune psychosis among individuals with 22q11DS (Table 4).

**Table 4.** Neuropsychiatric/developmental manifestation in 22q11.2 deletion syndrome

Author	Study design	Neuropsychiatric/developmental condition
Bassett et al., 2005 (Ref. 33)	Cross-sectional study	7 schizophrenia, 18 psychiatric disorders
Cancrini et al., 2014 (Ref. 46)	Cohort study	*n*/%: 143/83% neuropsychological manifestations, 115/70% speech and language delay, 87/53% psychomotor developmental delay, 99/65% learning difficulties, 14/11% behavioural abnormalities, 8/7% psychiatric disorders
Choi et al., 2005 (Ref. 47)	Cohort study	1 schizophrenia
Ciano-Petersen et al. 2021 (Ref. 74)	Case report	1 autoimmune psychosis
Crowley et al., 2022 (Ref. 48)	Cohort study	52% ADHD, 19% autism spectrum disorder, 15% seizures, 15% psychotic disorders
Davies et al., 2001 (Ref. 79)	Case report	2 developmental delay
DePiero et al., 1997 (Ref. 81)	Case report	1 learning disabilities
Giardino et al., 2014 (Ref. 52)	Cohort study	12 mental retardation,^a^ 4 seizures
Grinde et al., 2020 (Ref. 53)	Cross-sectional	43 neurodevelopmental disorders (ADHD, ADD, ASD and IDs); neuropsychiatric disorders (anxiety, major depressive disorder and psychosis); learning disabilities and developmental delay
Iijima et al., 2021 (Ref. 88)	Case report	‘learning disability’, ‘mentally retarded^a^’
Kawame et al., 2001 (Ref. 89)	Case report	1 schizophrenia, 1 seizure
Kotcher et al., 2022 (Ref. 55)	Cohort study	15% (30/197) Psychosis
Kratz et al., 2003 (Ref. 91)	Case report	Slowly developing neuropsychological disorder in association with cerebral atrophy of the brain
Nakada et al., 2013 (Ref. 94)	Case report	mild learning disability
Nissan et al., 2021 (Ref. 23)	Cohort study	1 mental disorder, 1 autism
Okiyama et al., 2005 (Ref. 96)	Case report	‘mental retardation^a^’
Ricci et al., 2018 (Ref. 28)	Cohort study	10/73 (13.7%) neuropsychiatric disorders
Soares et al., 2020 (Ref. 108)	Case report	schizophrenia
Stagi et al., 2010 (Ref. 64)	Cross-sectional + control group	schizophrenia
Ueda et al., 2017 (Ref. 110)	Case report	schizophrenia
Vergaelen et al., 2015 (Ref. 112)	Case report	1 ASD, 1 behavioural problem with oppositional behaviour, Impulse control disorder, 1 ADHD, 1 severe intellectual disability
Wahrmann et al., 2023 (Ref. 24)	Case–control study	93.2% neuropsychiatric disorders
Yousaf et al., 2021 (Ref. 115)	Case report	1 ADHD
Yu et al., 2022 (Ref. 66)	Cohort study	46 (52.87%) neuropsychiatric diseases

ADD: attention-deficit disorder; ADHD: attention-deficit/hyperactive disorder; ASD: autism spectrum disorder; ID: intellectual disability.

a Original text used the term ‘mental retardation’ and that the current preferred terminology is ‘intellectual disability’.

Out of the 82 studies reviewed, 65 studies included a blood work-up for autoimmunity, and 17 studies used medical records to retrieve information about autoimmune conditions, mostly retrospective studies. Seven studies were not primarily investigating autoimmune conditions but reported them as part of their findings.

**Figure 2.**
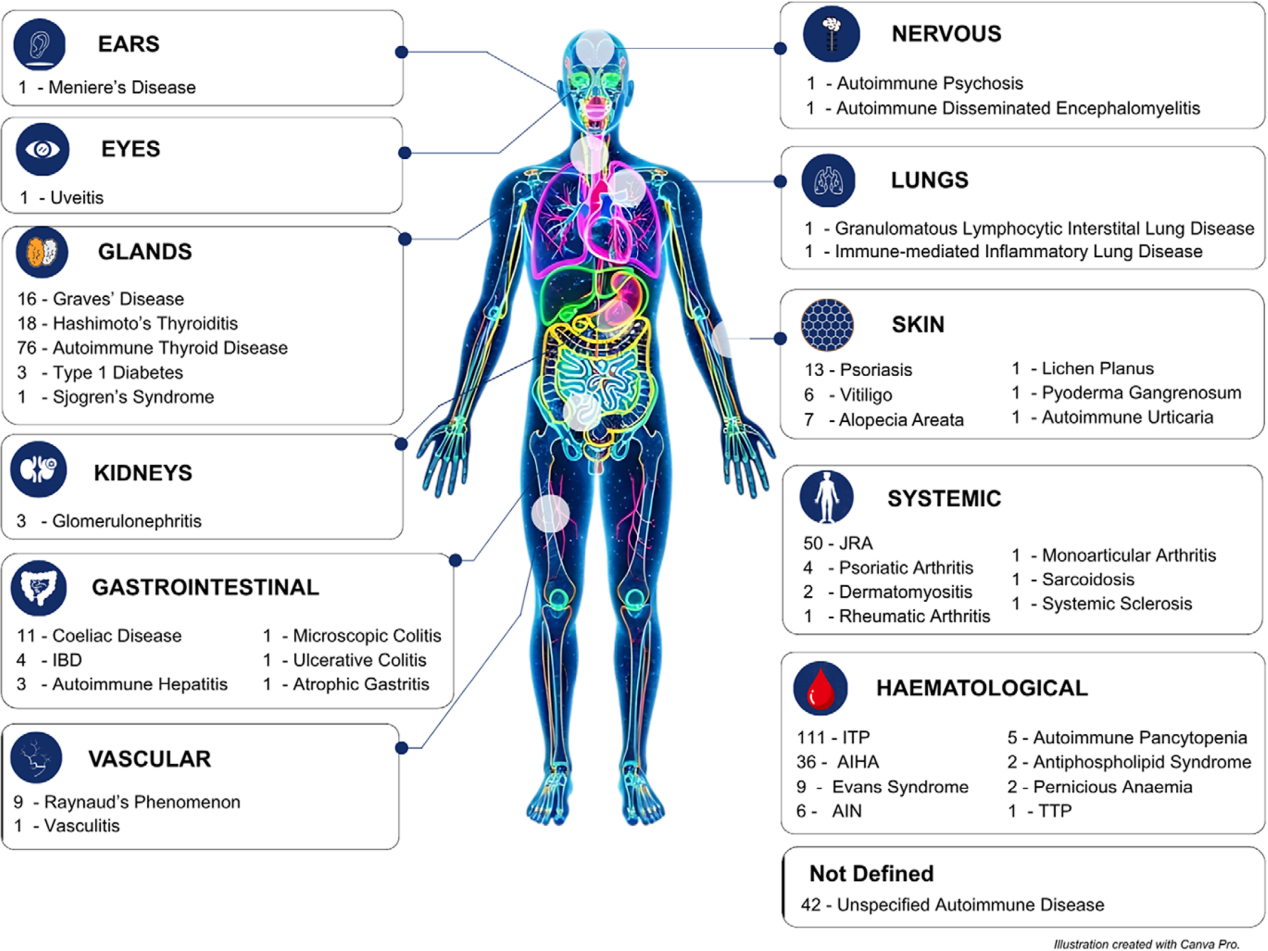
Overview of autoimmune diseases by organ/system in 22q11DS patients. *Note*: This figure illustrates the number of reported cases of autoimmune diseases categorised by the affected organ or system in individuals with 22q11DS. Each system is represented by a labelled icon, with specific autoimmune conditions and their corresponding case counts. The most frequently affected systems include the haematological and glandular systems. A total of 42 cases were classified as unspecified autoimmune diseases, reflecting papers that did not name specific autoimmune conditions but instead being generally present. Supplementary Table 2 also contains cellular and molecular functions of genes within the typically region in 22q11DS. AIHA: autoimmune haemolytic anaemia; AIN: autoimmune neutropenia; IBD: inflammatory bowel disease; ITP: immune thrombocytopenia; JRA: juvenile rheumatoid arthritis; TTP: thrombotic thrombocytopenic purpura.

Figure 2 shows an overview of the autoimmune diseases detected in our review. We identified 40 autoimmune diseases and disorders. For 42 cases identified in three studies (Refs 22–24), the autoimmune disease was not specified. Organ-specific autoimmune diseases were more frequently reported than systemic autoimmune diseases (Table 5). The most common autoimmune conditions in our review were immune thrombocytopenic purpura (ITP; 111 cases), and autoimmune thyroid diseases, which comprise Hashimoto’s and Graves’ disease (110 cases) but were sometimes not fully specified. In addition, autoimmune connective tissue diseases, particularly juvenile rheumatoid arthritis (JRA; 50 cases), were also common. Notably, 19 of these autoimmune conditions were rare and reported only once (in one individual with 22q11DS): autoimmune urticaria, autoimmune psychosis, atopic gastritis, acute disseminated encephalomyelitis, granulomatous lymphocytic interstitial lung disease, lichen planus, monoarticular arthritis, Meniere’s disease, pyoderma gangrenosum, rheumatoid arthritis, sarcoidosis, systemic sclerosis, Sjogren’s syndrome, thrombotic thrombocytopenic purpura, uveitis, ulcerative colitis, vasculitis, microscopic colitis and immune-mediated inflammatory lung disease. Nine autoimmune conditions were observed in five individuals or fewer (Figure 2).

**Table 5.** Summary of autoimmune diseases by organ/system in 22q11.2 deletion syndrome

Autoimmune disease	*n*	Author/year/country
Skin 29		
Psoriasis	13	Deshpande et al. 2021 (US), DiCesare et al. 2015 (Italy), Giardino et al. 2019 (UK), Jawad et al. 2001 (US), Legitimo et al. 2020 (Italy), Montin et al. 2019 (Italy), Nain et al. 2019 (Turkey), Tison et al. 2011 (US), Yi et al. 2023 (US)
Vitiligo	6	Deshpande et al. 2021 (US), Giardino et al. 2019 (UK), Jawad et al. 2001 (US), Ricci et al. 2018 (Italy), Tison et al. 2011 (US)
Alopecia Areata	7	Deshpande et al. 2021 (US), Giardino et al. 2019 (UK), Legitimo et al. 2020 (Italy), Ricci et al. 2018 (Italy)
Lichen planus	1	Pongpruttipan et al. 2012 (US)
Pyoderma gangrenosum	1	Comont et al. 2021 (France)
Autoimmune urticaria	1	Gennery et al. 2002 (UK)
Endocrine glands 113		
Graves’ Disease	16	Choi et al. 2005 (Korea), Crowley et al. 2022 (US), Lin et al. 2022 (Taiwan), Ricci et al. 2022 (Italy), Yu et al. 2022 (Taiwan), Brown et al. 2004 (UK), Hiroshima et al. 2022 (Japan), Iijima et al. 2021 (Japan), Kawame et al. 2001 (Japan), Kawamura et al. 2001 (Japan), Pong et al. 1985 (US), Ueda et al. 2017 (Japan)
Hashimoto’s thyroiditis	18	Hiroshima et al. 2022 (Japan), Nakada et al. 2013 (Japan), Choi et al. 2005 (Korea), Ricci et al. 2022 (Italy)
Autoimmune thyroid disease	76	Crowley et al. 2022 (US), Giardino et al. 2014 (Italy), Giardino et al. 2019 (UK), Mahe et al. 2019 (France), Ozen et al. 2021 (Turkey), Stagi et al. 2010 (Italy).
Type 1 diabetes	3	Elder et al. 2001 (US), Grinde et al. 2020 (Norway), Mahe et al. 2019 (France)
Exocrine glands 1		
Sjogren’s syndrome	1	Grinde et al. 2020 (Norway).
Gastrointestinal system 21		
Coeliac disease	11	DiCesare et al. 2015 (Italy), Digilio et al. 2003 (Italy), Giardino et al. 2014 (Italy), Kotcher et al. 2022 (US), Lima et al. 2011 (Norway), Ricci et al. 2018 (Italy)
Ulcerative colitis	1	Mahe et al. 2019 (France)
Atrophic gastritis	1	Grinde et al. 2020 (Norway)
IBD	4	Giardino et al. 2019 (UK), Jawad et al. 2001 (US)
Autoimmune hepatitis	3	DiCesare et al. 2015 (Italy), Lozano-Chinga et al. 2022 (US)
Microscopic colitis	1	Kotcher et al. 2022 (US)
Nervous System 2		
Autoimmune psychosis	1	Ciano-Petersen et al. 2021 (Spain)
Acute disseminated encephalomyelitis	1	Valenzise et al. 2014 (Italy)
Haematological system 172		
AIHA	36	Colarusso et al. 2010 (Italy), Damlaj et al. 2013 (Canada), Davies et al. 2003 (UK), DePiero et al. 1997 (US), Kratz et al. 2003 (Germany), Oliveras-Cordero et al. 2020 (US), Sakamoto et al. 2004 (Japan), Soldatou et al. 2013 (Greece), Yousaf et al. 2021, Björk et al. 2012 (Sweden), Cancrini et al. 2014 (Italy), Crowley et al. 2022 (US), Jawad et al. 2001 (US), Nain et al. 2019 (Turkey), Zama et al. 2021 (Italy), Giardino et al. 2019 (UK), Montin et al. 2019 (Italy)
AIN	6	Mahe et al. 2019 (France), Zama et al. 2021 (Italy), Tison et al. 2011 (US), Gu et al. 2023 (China), Kratz et al. 2003 (Germany), Lozano-Chinga et al. 2022
Autoimmune pancytopenia	5	Soldatou et al. 2013 (Greece), Bruno et al. 2002 (France), Yu et al. 2022 (Taiwan), Ozen et al. 2021 (Turkey)
Antiphospholipid syndrome	2	Chang et al. 2006 (US), Nucera et al. 2006 (Italy)
Evans syndrome	9	Oliveras-Cordero et al. 2020 (US), Soldatou et al. 2013 (Greece), Colarusso et al. 2010 (Italy), Conti et al. 2022 (Italy), Davies et al. 2003 (UK), Kratz et al. 2003 (Germany), Ricci et al. 2018 (Italy), Gennery et al. 2002 (UK)
ITP	111	Gennery et al. 2002 (UK), Giardino et al. 2019 (UK), Montin et al. 2019 (Italy), Zama et al. 2021 (Italy), Deshpande et al. 2021 (US), Levy et al. 1997 (France), Lozano-Chinga et al. 2022 (US), Oliveras-Cordero et al. 2020 (US), Soldatou et al. 2013 (Greece), Chang et al. 2006 (US), Colarusso et al. 2010 (Italy), Conti et al. 2022 (Italy), Comont et al. 2021 (France), Damlaj et al. 2013 (Canada), Davies et al. 2003 (UK), DePiero et al. 1997 (US), Hernandez-Nieto et al. 2011 (Mexico), Kratz et al. 2003 (Germany), Levy et al. 1997 (France), Ozen et al. 2021 (Turkey), Yu et al. 2022 (Taiwan), Bjork et al. 2012 (Sweden), Crowley et al. 2022 (US), DiCesare et al. 2015 (Italy), Klocperk et al. (2018), Mahe et al. 2019 (France)
Pernicious anaemia	2	Grinde et al. 2020 (Norway), Lima et al. 2011 (Norway)
Thrombotic thrombocytopenic purpura	1	Soares et al. 2020 (Brazil)
Vascular system 10		
Vasculitis	1	Gennery et al. 2002 (UK)
Raynaud’s phenomenon	9	Giardino et al. 2019 (UK), Lima et al. 2011 (Norway), Gennery et al. 2002 (UK), Pereira et al. 2003 (Netherlands)
Kidney 3		
Glomerulonephritis	3	Arkush et al. 2019 (Israel), Chang et al. 2006 (US), Crowley et al. (2022) (US)
Lung 2		
Granulomatous lymphocytic interstitial lung disease	1	Soares et al. 2020 (Brazil)
Immune-mediated inflammatory lung disease	1	Giardino et al. 2019 (UK)
Eyes 1		
Uveitis	1	Gottlieb et al. 2010 (US)
Ears 1		
Meniere’s disease	1	Choi et al. 2021 (Japan)
Systemic diseases 60		
Dermatomyositis	2	Okiyama et al. 2005 (UK), Giardino et al. 2019 (UK)
JRA	50	Jawad et al. 2001 (US), Sullivan et al. 1997 (US), Rasmussen et al. 1996 (US), Verloes et al. 1998 (Belgium), Desphande et al. 2021 (US), Montin et al. 2019 (Italy), Pelkonen et al. 2002 (Finland), Sestan et al. 2020 (US), Salehzadeh et al. 2014 (Iran), Mahe et al. 2019 (France), Nain et al. 2019 (Turkey)
Psoriatic arthritis	4	Lima et al. 2011 (Norway), Giardino et al. 2014 (Italy), Vergaelen et al. 2015 (Belgium), Yi et al. 2023 (US)
Rheumatic arthritis	1	Jawad et al. 2001 (US)
Monoarticular arthritis	1	Tison et al. 2011 (US)
Sarcoidosis	1	Grinde et al. 2020 (Norway)
Systemic sclerosis	1	Deshpande et al. 2021 (US)
Autoimmune disease not specified 42		
Autoimmune diseases	42	McLean-Tooke et al. 2008 (UK), Nissan et al. 2021 (Israel), Wahrmann et al. 2023 (Finland)

AIHA: autoimmune haemolytic anaemia; AIN: autoimmune neutropenia; IBD, inflammatory bowel disease; ITP, immune thrombocytopenic purpura; JRA, juvenile rheumatoid arthritis.

Some articles reported autoantibodies in 22q11DS (Tables 2 and 3). Two studies included in our review did not report on autoimmune conditions; rather, they reported autoantibodies (Refs 25, 26).

### Skin

Twenty-one studies conducted in France, the United States, Italy, the United Kingdom and Turkey reported autoimmune conditions related to the skin among individuals with 22q11DS (Table 5). Psoriasis (*n* = 13) was the most common autoimmune condition affecting the skin, reported in nine articles. Other conditions included vitiligo (*n* = 6) reported in five articles, alopecia areata (*n* = 7) reported in four articles and autoimmune urticaria (*n* = 1) reported in one article. Lichen planus, pyoderma gangrenosum and psoriasis were documented as case reports. No autoantibodies related to the skin were reported.

### Endocrine and exocrine glands

Twenty-four studies conducted in the United Kingdom, Japan, the United States, Korea, Taiwan, Italy, Norway, France and Turkey reported three autoimmune conditions involving the endocrine glands: autoimmune thyroid disease (comprising Graves’ disease and Hashimoto’s thyroiditis) and type 1 diabetes. Collectively, there were 110 cases of autoimmune thyroid disease reported. Three cases of type 1 diabetes were reported in three studies (Norway, the United States and France). Sjogren’s syndrome, the only autoimmune condition involving exocrine glands in our review, was reported in a study conducted in Norway (Table 5).

### Gastrointestinal

Thirteen studies conducted in the United States, Norway, Italy, France and the United Kingdom reported autoimmune conditions involving the gastrointestinal system among individuals with 22q11DS. Coeliac disease (*n* = 11) was the most common gastrointestinal autoimmune condition, reported in six studies. Two studies reported inflammatory bowel disease (IBD; *n* = 4), although the type of IBD was not specified. However, one case of ulcerative colitis was reported in an article conducted in France. Autoimmune hepatitis (*n* = 3) was reported in three studies, and atrophic gastritis (*n* = 1) was reported in one article.

### Neurological system

Two studies conducted in Spain and Italy, respectively, reported autoimmune psychosis (*n* = 1) and acute disseminated encephalomyelitis (*n* = 1) as case reports.

### Haematological system

Sixty-four studies reported autoimmune conditions involving the haematological system. ITP was the most common autoimmune condition in our review, accounting for 64.5% of haematological cases. A study conducted in the United Kingdom (Ref. 27) reported that autoimmune cytopenia was the most common autoimmune condition in their cohort. Other conditions involving the haematological system include autoimmune haemolytic anaemia (*n* = 36), reported in 17 studies; autoimmune neutropenia (*n* = 6), reported in 6 studies; autoimmune pancytopenia (*n* = 5), reported in 4 studies; antiphospholipid syndrome (*n* = 2), reported in 2 studies; Evans syndrome (*n* = 9), reported in 6 studies; pernicious anaemia (*n* = 2), reported in 2 studies; and thrombotic thrombocytopenic purpura (*n* = 1), reported in 1 article. These studies were conducted in Italy, the United Kingdom, the United States, Canada, Sweden, the Czech Republic, Norway, France, Turkey, Taiwan, Kuwait, China, Mexico, Germany, Puerto Rico, Israel, Qatar, Greece and Brazil.

### Vascular system

Five studies reported autoimmune conditions related to the vascular system. Raynaud’s phenomenon (*n* = 9), reported in four studies, and vasculitis (*n* = 1), reported in one article, were the two autoimmune conditions identified in our review involving the vascular system. These studies were conducted in Europe (the United Kingdom, Norway and the Netherlands), with two Raynaud’s phenomenon cases documented as case reports.

### Kidney

Three studies conducted in the United States and Israel reported glomerulonephritis (*n* = 3), with two of the three studies being reported as case reports. No other autoimmune conditions involving the kidney were reported.

### Eyes/ears

Uveitis (*n* = 1), an autoimmune condition involving the eye, was reported as a case report in an article conducted in the United States, and Meniere’s disease (*n* = 1), an autoimmune condition involving the ears, was also reported as a case report in an article from Japan.

### Lungs

Granulomatous lymphocytic interstitial lung disease (*n* = 1) and immune-mediated inflammatory lung disease (*n* = 1) were reported in one article each.

### Systemic autoimmune rheumatic diseases

Eleven studies reported seven systemic autoimmune conditions. JRA (*n* = 50) was the most common systemic autoimmune condition, reported in 11 studies conducted in Europe (Sweden, Italy, the United Kingdom, France, Turkey, Finland and Belgium) and one case conducted in Iran. Fourteen of these studies were case reports. Psoriatic arthritis (*n* = 4) was reported in four studies conducted in Italy, Norway Belgium and the United States. Dermatomyositis (*n* = 2) was reported in two studies conducted in the United Kingdom, whereas rheumatoid arthritis (*n* = 1), monoarticular arthritis (*n* = 1), sarcoidosis (*n* = 1) and systemic sclerosis (*n* = 1) were reported in one article each conducted in the United States and Norway.

### Quality assessment

The quality of the included studies (cohort, case–control and cross-sectional) was assessed using the NOS. This scale measures study quality based on selection, comparability and the ascertainment of exposure/outcome. The scale has an eight-item instrument for cohort or case–control studies, and the rating system ranges from 0 to 9 stars. Studies with scores ≥7 points represented low risk of bias (high quality), 4–6 points as moderate risk of bias (intermediate quality) and those with a score of 1–3 points as high risk of bias (low quality).

Two of the 34 studies had a low risk of bias with a score of 7 (high quality). Twenty-three studies scored 4–6, showing moderate risk of bias and, thus, intermediate quality. Nine studies had a high risk of bias scoring between 1 and 3 points (Table 6).

**Table 6.** Newcastle–Ottawa Quality Assessment Scale for cohort, case–control and cross-sectional studies

Author	Number of stars in each domain			
	Selection	Comparability	Outcome/exposure^a^	Study quality
Bassett et al., 2005 (Ref. 33)	2	0	1	Low
Björk et al., 2012 (Ref. 45)	3	0	1	Moderate
Cancrini et al., 2014 (Ref. 46)	3	0	3	Moderate
Choi et al., 2005 (Ref. 47)	3	0	3	Moderate
Crowley et al., 2022 (Ref. 48)	3	1	2	Moderate
Deshpande et al., 2021 (Ref. 49)	2	1	1	Moderate
DiCesare et al., 2015 (Ref. 50)	2	1	1	Moderate
Digilio et al., 2003 (Ref. 51)	2	0	2	Moderate
Gennery et al., 2002 (Ref. 10)	2	0	2	Moderate
Giardino et al., 2014 (Ref. 52)	2	0	1	Low
Giardino et al., 2019 (Ref. 27)	3	1	2	Moderate
Grinde et al., 2020 (Ref. 53)	1	1	1	Low
Jawad et al., 2001 (Ref. 54)	2	1	1	Moderate
Klocperk et al., 2018 (Ref. 21)	2	0	1	Low
Kotcher et al., 2022 (Ref. 55)	2	1	2	Moderate
Legitimo et al., 2020 (Ref. 56)	3	1	1	Moderate
Lima et al., 2011 (Ref. 57)	2	1	1	Moderate
Lin et al., 2022 (Ref. 58)	2	1	2	Moderate
Mahe et al., 2019 (Ref. 59)	2	1	2	Moderate
McLean-Tooke et al., 2008 (Ref. 60)	3	1	1	Moderate
Montin et al., 2019 (Ref. 31)	3	0	2	Moderate
Nain et al., 2019 (Ref. 61)	3	1	2	Moderate
Nissan et al., 2021 (Ref. 23)	2	0	1	Low
Ongen et al., 2023 (Ref. 26)	2	0	1	Low
Ozen et al., 2021 (Ref. 62)	2	0	1	Low
Ricci et al., 2018 (Ref. 63)	3	1	1	Moderate
Ricci et al., 2022 (Ref. 28)	4	1	3	High
Sedivá et al., 2005 (Ref. 25)	2	0	1	Low
Stagi et al., 2010 (Ref. 64)	3	1	1	Moderate
Sullivan et al., 1997 (Ref. 38)	2	0	3	Moderate
Tison et al., 2011 (Ref. 65)	2	1	3	Moderate
Wahrmann et al., 2023 (Ref. 24)	4	1	2	High
Yu et al., 2022 (Ref. 66)	2	0	3	Moderate
Zama et al., 2021 (Ref. 67)	1	0	1	Low

a Outcome for cohort and cross-sectional studies; exposure for case–control study.

Of the studies with low quality, the percentage of 22q11DS patients with autoimmunity was estimated to be 2.1%–29.4%. For studies with moderate quality, autoimmunity was estimated to be present in 0.7%–47% of 22q11DS patients. For the two studies with high quality, Ricci et al. (Ref. 28) reported that 21.9% had autoimmunity. The specific autoimmune diseases reported were Hashimoto’s thyroiditis and Grave’s disease. For Wahrmann et al. (Ref. 24), 29.6% had autoimmunity. However, the specific autoimmune diseases were not reported.

## Overview and discussion

We performed a systematic analysis of the diverse evidence and heterogeneity of autoimmune diseases in 22q11DS. Autoimmunity is recognised clinically as a complication of 22q11DS, and there have been several excellent reviews (Refs 22, 29, 30). However, there has not been a systematic, comprehensive and unbiased evaluation of the evidence. We performed this review utilising a transparent and rigorous methodology to thoroughly review and assess 82 studies across a variety of designs.

Collectively, the reviewed literature was comprised of diverse and inconsistent study designs. A large number of studies were case reports or case series. Many studies did not describe a thorough rationale for the selection of patients or research subjects. Thus, selection bias is likely, and a pooled prevalence estimate could be unreliable because many studies lacked a clear quantification of the total population under consideration at a given time. Nevertheless, our systematic approach provides the framework for future studies, and we identify major gaps in literature.

Our analysis suggested several facets of autoimmunity in 22q11DS, including the hierarchy of the specific organ systems involved. Haematological autoimmune findings were the most common, reported in 64 studies. These included ITP, which comprised 24% of the diagnosed autoimmune diseases, autoimmune haemolytic anaemia (8%) and Evans syndrome (2%). A prior study also suggested that blood cells are the most common target of autoimmunity in 22q11DS (Ref. 31). This same study reported that specific T- and B-cell immunophenotypic anomalies predicted subsequent haematological autoimmunity (Ref. 31).

Autoimmune thyroid disease was also common, accounting for 24% of the overall cases of autoimmune diseases we counted. The thyroid is often affected by autoimmunity in the general population (Ref. 28), and we found that both Graves’ disease and Hashimoto’s thyroiditis were well represented among the cases. In a longitudinal study of children with 22q11DS, 16 of the 73 patients (21.9%) developed autoimmune thyroid disease before 18 years of age, and 20.5% developed Hashimoto’s thyroiditis, whereas only 1.4% developed Graves’ disease (Ref. 28). We found that many studies did not specify the type of autoimmune thyroid disease detected.

Various gastrointestinal autoimmune diseases were identified in our study, with coeliac disease being the most common. This may be relevant to the psychiatric manifestations of 22q11DS, because coeliac disease is associated with schizophrenia (Ref. 32), and individuals with 22q11DS have an extremely high rate of schizophrenia (Refs 33, 34).

However, only 24 papers reported on neuropsychiatric or neurodevelopmental conditions among individuals with 22q11DS. These disorders included autism spectrum disorder, developmental delay, attention-deficit/ hyperactive disorder and schizophrenia. This is not surprising because these disorders are known to be exceptionally common in 22q11DS (Ref. 35), but we could not calculate whether autoimmunity impacted their prevalence or presentation because the relevant studies reviewed are not conductive to this analysis (see above). Hypocalcemia, caused by hypoparathyroidism, is a well-established complication of 22q11DS and has been reported to be associated with haematological autoimmunity as well as neurodevelopmental delay (Refs 31, 36).

Autoimmune diseases of the skin were detected in several different studies in Europe and the United States, with psoriasis, vitiligo and alopecia areata being the most common. Autoimmunity conditions of the eyes, lung and kidney were also reported, although these were relatively rare. Thus, multiple organs are affected by autoreactive immune mechanisms in 22q11DS.

Systemic autoimmune conditions were also common, which underscores the need for diverse specialists in treating and diagnosing patients with 22q11DS (Ref. 37). An increased rate of JRA in 22q11DS was suggested over 25 years ago (Ref. 38), and this is one of several skeletal disorders affecting patients with 22q11DS (Ref. 39). We also found evidence for autoimmune conditions of the vascular system (i.e., Raynaud’s phenomenon and vasculitis).

Genes that are in the TDR participate in a wide variety of cellular and molecular processes, and we would expect many of these processes to be affected by the underexpression of these genes. This could interact with autoimmunity in several ways. For example, thyroid dysfunction is known to occur in 22q11DS due to the underdevelopment of the third and fourth pharyngeal pouches (Ref. 40), and an autoimmune attack on this gland could exacerbate or complicate hypothyroidism. Another possible mechanism involves the underexpression of 22q11DS genes in immune cells or in the target organ of autoimmunity This could result in the breaking of immunotolerance by abnormalities in immune receptors, cell trafficking, or apoptosis in immune tissues or the target organ. Whether a given individual with 22q11DS develops an autoimmune disease may depend on their human leukocyte antigen type, genetic polymorphisms outside the TDR and their particular environmental exposures.

The confidence in the estimated frequency of autoimmune diseases in 22q11DS is limited because of the inclusion of many studies with methodological limitations. This is reflected in the low and moderate scores on the NOS scale, mostly identified in the selection and comparability domains. Some studies did not adjust for confounders, whereas others did not report on the comparability of the groups included. Thus, these weaknesses lead to a potential for bias, indicating that the observed frequency of autoimmune diseases in 22q11DS is imprecise.

Comanagement of patients with 22q11DS in a multidisciplinary setting is warranted. This approach addresses the diverse medical and psychosocial needs of patients. Ideally, clinics specialising in 22q11DS will be arranged so that a patient can visit multiple specialists (e.g., haematologist, immunologist, endocrinologist, cardiologist, speech therapist and psychologist) on the same day based on their particular needs. For example, such a clinic might operate once a month with all specialists being available that day.

### Gaps and limitations

Our review identified several gaps in the clinical and research evidence connecting 22q11DS to autoimmunity. Collection of the articles included in this review was completed by October 2023, thereby missing newer reports. The majority of the studies (59%) were case reports. Among the rest of the studies, the rationale for the selection of the individuals included was often not clearly stated. Most studies were from Europe, with most of the rest being from either North America or Asia. There was substantial heterogeneity in both the age of the patients and the criteria or methodology used for diagnosis. Many of the reports of diseases known to be caused by autoantibodies did not perform autoantibody testing. A finding in our quality metrics for case–control, cohort and cross-sectional studies indicated a wide range of quality and potential biases. Nonetheless, the reliance on the NOS can involve some subjective interpretation, which was minimised through independent assessment by two reviewers. However, a degree of uncertainty remains.

To maximise the number and type of studies included in our review, we used a broad definition of autoimmune disease based on criteria put forth by the NIH (United States). It is possible that some of the autoimmune conditions that we captured may have had a more complex aetiology besides (or in addition to) autoimmunity.

Although a meta-analysis would be desirable, this systematic review found that the data are not very conducive to meta-analysis. There was substantial methodological and statistical heterogeneity even among the case–control and cohort studies. Different diagnostic criteria and outcome measures were used, and the ambiguity as to the source of the sample and population argues further against attempting to pool studies for meta-analysis until more primary data become available.

Despite these limitations, this review fills a major gap by our in-depth analysis, which elucidates the variety of autoimmune diseases detected in 22q11DS, and their comorbidity with neuropsychiatric disorders. The benefits of shedding light on this important topic outweigh the limitations.

### Expert and topical summary

This systematic review confirmed that autoimmunity is a component of 22q11DS. However, there is a major need for a thorough epidemiological evaluation of autoimmune prevalence across organ systems, as well as to determine relevant comorbidities and disease course in ethnically and geographically diverse populations affected by 22q11DS. Montin et al. (Ref. 31) estimated the prevalence of autoimmunity among 358 persons with 22q11DS to be 24%. The current review broadens the view of 22q11DS and demonstrates the diverse organ systems affected.

Neuropsychological disorders are a very common feature of 22q11DS (Refs 33, 34, 41, 42), and an autoimmune aetiopathogenesis is possible in some of these cases (Ref. 43); however, we found that few of the case–control and cohort studies reported expressly on psychological or specific neurodevelopmental conditions. This emphasises the need for a deeper investigation of the pathophysiological connection between autoimmunity and neuropsychiatric manifestations of 22q11DS.

There has been substantial progress in the last two decades on understanding the immunology of autoreactivity in 22q11DS, which is also informed by researching other inborn errors of immunity (Ref. 30). This immunological work holds promise for new therapies, and relevant mouse models of 22q11DS can help inform both pathophysiology and aid in the development and testing of therapeutic modalities (Ref. 44).

An ensemble of specialists work to improve the lives of persons with 22q11DS. The work of all these specialties will benefit from a better understanding of the wide spectrum of autoimmune manifestations in this disorder.

## Supporting information

10.1017/erm.2026.10031.sm001 Ogunsola et al. supplementary material 1 Ogunsola et al. supplementary material

10.1017/erm.2026.10031.sm002 Ogunsola et al. supplementary material 2 Ogunsola et al. supplementary material

## Supplementary material

The supplementary material for this article can be found at http://doi.org/10.1017/erm.2026.10031.
