# Supplementary material for: A Systematic Review of Autoimmunity in 22q11.2 Deletion Syndrome
Source: Expert Rev Mol Med. 2026 Jan 8;28:e12. doi: 10.1017/erm.2026.10031 (PMC13148428; doi:10.1017/erm.2026.10031)
Supplement: Ogunsola et al. supplementary material 1 — Ogunsola et al. supplementary material [file S1462399426100313sup001.docx]

# Supplementary table 1: Search Strategy for autoimmunity in 22q11.2 Deletion Syndrome

| PubMed 10/04/2023 | (autoimmun* or auto-immune or auto-immunity or autoantibod* or auto-antibod* or Achalasia or "Addison* disease" or "Addison Disease"[Mesh] or "Still* disease" or "Arthritis, Juvenile"[Mesh] or Agammaglobulinemia or "Alopecia areata" or "Alopecia Areata"[Mesh] or Amyloidosis "Ankylosing spondylitis" OR "Spondylitis, Ankylosing"[Mesh] or “Anti-GBM nephritis" or "anti tbm nephritis" OR "Anti-Glomerular Basement Membrane Disease"[Mesh] or "Antiphospholipid syndrome" Or "Antiphospholipid Syndrome"[Mesh] or "Autoimmune angioedema" or "Autoimmune dysautonomia" or "Autoimmune encephalitis" or "Autoimmune hepatitis" or "Autoimmune urticaria" or "Chronic Urticaria"[Mesh] or "Axonal neuropathy" or "Neuronal neuropathy" or "Balo disease" or "Diffuse Cerebral Sclerosis of Schilder"[Mesh] or "Behcet* disease" or "Behcet Syndrome"[Mesh] or "Benign mucosal pemphigoid" or "Bullous pemphigoid" or "Pemphigoid, Benign Mucous Membrane"[Mesh] or "Castleman disease" or "Castleman Disease"[Mesh] or "Celiac disease" or "Celiac Disease"[Mesh] or "Chagas disease" or "Chagas Disease"[Mesh] or "Chronic inflammatory demyelinating polyneuropathy" or "Polyradiculoneuropathy, Chronic Inflammatory Demyelinating"[Mesh] or "Chronic recurrent multifocal osteomyelitis" or "Chronic recurrent multifocal osteomyelitis" [Supplementary Concept] or "Churg-Strauss syndrome" or "Churg-Strauss Syndrome"[Mesh] or "Eosinophilic granulomatosis" or "Cicatricial pemphigoid" or "Cogan* syndrome" or "Cogan Syndrome"[Mesh] or "Cold agglutinin disease" or "Complex regional pain syndrome" or "Complex Regional Pain Syndromes"[Mesh] or "sympathetic dystrophy" or "Congenital heart block" or "Congenital heart block" [Supplementary Concept] or "Coxsackie myocarditis" or "CREST syndrome" or "CREST Syndrome"[Mesh] or "Crohn* disease" or "Crohn Disease"[Mesh] or "Dermatitis herpetiformis" or "Dermatitis Herpetiformis"[Mesh] or Dermatomyositis or "Devic* disease" or "Neuromyelitis Optica"[Mesh] or "Dressler* syndrome" or “Donath-Landsteiner hemolytic anemia” [Supplementary Concept] or Endometriosis or "Eosinophilic esophagitis" or "Eosinophilic fasciitis" or "Eosinophilic Esophagitis"[Mesh] or "Erythema nodosum" or "Erythema Nodosum"[Mesh] or "Essential mixed cryoglobulinemia" or "Evans syndrome" or Evans Syndrome [Supplementary Concept] or Fibromyalgia or "Fibrosing alveolitis" or "Giant cell arteritis" or "Giant Cell Arteritis"[Mesh] or "temporal arteritis” or "Giant cell myocarditis" or Glomerulonephritis or "Goodpasture* syndrome" or Granulomatosis or "Graves* disease" or "Graves Disease"[Mesh] or Guillain-Barre or "Hashimoto* thyroiditis" or "Hashimoto Disease"[Mesh] or "Hemolytic anemia" or "Anemia, Hemolytic, Autoimmune"[Mesh] or "Henoch-Schonlein purpura" or "IgA Vasculitis"[Mesh] or "Herpes gestationis" or "Pemphigoid Gestationis"[Mesh] or "Hidradenitis suppurativa" or "Hidradenitis Suppurativa"[Mesh] or "acne inversa" or "IgA nephropathy" or "IgG4-related sclerosing disease" or "Immune thrombocytopenic purpura" or "Purpura, Thrombocytopenic, Idiopathic"[Mesh] or "Interstitial cystitis" or "Cystitis, Interstitial"[Mesh] or "Juvenile arthritis" or "Juvenile diabetes" or "type I diabetes" or "Diabetes Mellitus, Type 1"[Mesh] or "Kawasaki disease" or "Mucocutaneous Lymph Node Syndrome"[Mesh] or "Lambert-Eaton syndrome" or "Lichen planus" or "Lichen Planus"[Mesh] or "Lichen sclerosus" or "Ligneous conjunctivitis" or "Linear IgA disease" or "Linear IgA Bullous Dermatosis"[Mesh] or Lupus or "Lyme disease" or "Lyme Disease"[Mesh] or "Meniere* disease" or "Meniere Disease"[Mesh] or "Microscopic polyangiitis" or "Microscopic Polyangiitis"[Mesh] or "Mixed connective tissue disease" or "Mixed Connective Tissue Disease"[Mesh] or "Mucha-Habermann disease" or "Pityriasis Lichenoides"[Mesh] or "Multifocal motor neuropathy" or "Multiple sclerosis" or "Multiple Sclerosis"[Mesh] or "Myasthenia gravis" or "Myasthenia Gravis"[Mesh] "Myelin oligodendrocyte glycoprotein" or "Myelin-Oligodendrocyte Glycoprotein"[Mesh] or Myositis or Narcolepsy or Neutropenia or "Ocular cicatricial pemphigoid" or "Optic neuritis" or "Optic Neuritis"[Mesh] or "Palindromic rheumatism" or "Palindromic rheumatism" [Supplementary Concept] or PANDAS or "Pediatric autoimmune neuropsychiatric disorders" or "Pediatric Autoimmune Neuropsychiatric Disorders Associated with Streptococcal infections" [Supplementary Concept] or "Paraneoplastic cerebellar degeneration" or "Paraneoplastic Cerebellar Degeneration"[Mesh] or "Paroxysmal nocturnal hemoglobinuria" or "Hemoglobinuria, Paroxysmal"[Mesh] or "Pars planitis" or "Pars Planitis"[Mesh] or “peripheral uveitis” or "Parsonage-Turner syndrome" or "Brachial Plexus Neuritis"[Mesh] or Pemphigus or "Peripheral neuropathy" or "Perivenous encephalomyelitis" or "Pernicious anemia" or "Anemia, Pernicious"[Mesh] or "POEMS syndrome" or "POEMS Syndrome"[Mesh] or "Polyarteritis nodosa" or "Polyarteritis Nodosa"[Mesh] or "Polyglandular syndromes" or "Polymyalgia rheumatica" or "Polymyalgia Rheumatica"[Mesh] or Polymyositis or "Postmyocardial infarction syndrome" or "Postpericardiotomy syndrome" or "Postpericardiotomy Syndrome"[Mesh] or "Primary biliary cholangitis" or "Primary sclerosing cholangitis" or "Liver Cirrhosis, Biliary"[Mesh] or "Progesterone dermatitis" or Autoimmune progesterone dermatitis [Supplementary Concept] or "Progressive hemifacial atrophy" or "Parry Romberg syndrome" or "Facial Hemiatrophy"[Mesh] or Psoriasis or "Psoriatic arthritis" or "Arthritis, Psoriatic"[Mesh] or "Pure red cell aplasia" or "Red-Cell Aplasia, Pure"[Mesh] or "Pyoderma gangrenosum" or "Pyoderma Gangrenosum"[Mesh] or Raynaud* or "Reactive arthritis" or "Arthritis, Reactive"[Mesh] or "Relapsing polychondritis" or "Polychondritis, Relapsing"[Mesh] or "Restless legs syndrome" or "Restless Legs Syndrome"[Mesh] or "Retroperitoneal fibrosis" or "Retroperitoneal Fibrosis"[Mesh] or "Rheumatic fever" or "Rheumatic Fever"[Mesh] or "Rheumatoid arthritis" or "Arthritis, Rheumatoid"[Mesh] or Sarcoidosis or "Schmidt syndrome" or "autoimmune polyendocrine syndrome" OR "Polyendocrinopathies, Autoimmune"[Mesh] or Scleritis or Scleroderma or "Sjogren* Disease" or "Stiff person syndrome" OR "Stiff-Person Syndrome"[Mesh] or "Susac* syndrome" OR "Susac Syndrome"[Mesh] or "Sympathetic ophthalmia" OR "Ophthalmia, Sympathetic"[Mesh] or "Takayasu* arteritis" or "Takayasu Arteritis"[Mesh] OR "Thrombocytopenic purpura" or "Thrombotic thrombocytopenic purpura" OR "Purpura, Thrombocytopenic"[Mesh] or "Thyroid eye disease" OR "Graves Ophthalmopathy"[Mesh] or "Tolosa-Hunt syndrome" OR "Tolosa-Hunt Syndrome"[Mesh] or "Transverse myelitis" OR "Myelitis, Transverse"[Mesh] or "Ulcerative colitis" OR "Colitis, Ulcerative"[Mesh] or "Undifferentiated connective tissue disease" OR "Undifferentiated Connective Tissue Diseases"[Mesh] or Uveitis or Vasculitis or Vitiligo or "Vogt-Koyanagi-Harada disease" OR "Uveomeningoencephalitic Syndrome"[Mesh]) AND    ("DiGeorge Syndrome"[Mesh] OR "Digeorge Syndrome-Velocardiofacial Syndrome Complex 2" [Supplementary Concept] OR "Digeorge Syndrome-Velocardiofacial Syndrome Complex 2" [Supplementary Concept] or “DiGeorge Syndrome” OR “Di George Syndrome” OR “DiGeorge Sequence” OR “Di George Sequence” OR “DiGeorge Anomaly” OR “Di George Anomaly” OR DiGeorge OR “Di George” OR Di-George OR “Pharyngeal Pouch syndrome” OR “Autosomal Dominant Opitz G-Bbb Syndrome” OR “Autosomal Dominant Opitz G Bbb Syndrome” OR “Thymic Aplasia Syndrome” OR Catch22 OR “catch 22” OR “Hypoplasia of Thymus” OR “thymus hypoplasia” OR "parathyroid* hypoplasia" OR “Velocardiofacial Syndrome” OR “Syndrome, Velocardiofacial” OR “VCF Syndrome” OR “Syndrome, VCF” OR “Velo-Cardio-Facial Syndrome” OR “syndrome, Velo-Cardio-Facial” “Velo Cardio Facial Syndrome” OR “Syndrome, Velo-Cardio-Facial” OR “Sedlackova Syndrome” OR “Syndrome, Sedlackova” OR Shprintzen OR 22q11.2DS OR 22q11.2* OR “Conotruncal Anomaly” OR CTAF OR “Takao syndrome” OR “Cayler cardiofacial” OR “Cayler syndrome” OR TBX1)  Limited to English |
| --- | --- |
| **Web of Science 10/04/2023** | autoimmun* or auto-immune or auto-immunity or autoantibod* or auto-antibod* or Achalasia or "Addison* disease" or "Still* disease" or Agammaglobulinemia or "Alopecia areata" or Amyloidosis "Ankylosing spondylitis" or “Anti-GBM nephritis" or "anti tbm nephritis" OR "Anti-Glomerular Basement Membrane Disease" or "Antiphospholipid syndrome" or "Autoimmune angioedema" or "Autoimmune dysautonomia" or "Autoimmune encephalitis" or "Autoimmune hepatitis" or "Autoimmune urticaria" or "Chronic Urticaria" or "Axonal neuropathy" or "Neuronal neuropathy" or "Balo disease" or "Diffuse Cerebral Sclerosis of Schilder" or "Behcet* disease" or "Behcet Syndrome" or "Benign mucosal pemphigoid" or "Bullous pemphigoid" or "Castleman disease" or "Celiac disease" or "Chagas disease" or "Chronic inflammatory demyelinating polyneuropathy" or "Chronic recurrent multifocal osteomyelitis" or "Churg-Strauss syndrome" or "Eosinophilic granulomatosis" or "Cicatricial pemphigoid" or "Cogan* syndrome" or "Cold agglutinin disease" or "Complex regional pain syndrome*" or "sympathetic dystrophy" or "Congenital heart block" or "Coxsackie myocarditis" or "CREST syndrome" or "Crohn* disease" or "Dermatitis herpetiformis" or Dermatomyositis or "Devic* disease" or "Neuromyelitis Optica” or "Dressler* syndrome" or “Donath-Landsteiner hemolytic anemia” or Endometriosis or "Eosinophilic esophagitis" or "Eosinophilic fasciitis" or "Erythema nodosum" or "Essential mixed cryoglobulinemia" or "Evans syndrome" or Fibromyalgia or "Fibrosing alveolitis" or "Giant cell arteritis" or "temporal arteritis” or "Giant cell myocarditis" or Glomerulonephritis or "Goodpasture* syndrome" or Granulomatosis or "Graves* disease" or Guillain-Barre or "Hashimoto* thyroiditis" or "Hashimoto Disease" or "Hemolytic anemia" or "Henoch-Schonlein purpura" or "IgA Vasculitis" or "Herpes gestationis" or "Pemphigoid Gestationis" or "Hidradenitis suppurativa" or "acne inversa" or "IgA nephropathy" or "IgG4-related sclerosing disease" or "Immune thrombocytopenic purpura" or "Interstitial cystitis" or "Juvenile arthritis" or "Juvenile diabetes" or "type I diabetes" or "Diabetes Mellitus, Type 1" or "Kawasaki disease" or "Mucocutaneous Lymph Node Syndrome" or "Lambert-Eaton syndrome" or "Lichen planus" or "Lichen sclerosus" or "Ligneous conjunctivitis" or "Linear IgA disease" or Lupus or "Lyme disease" or "Meniere* disease" or "Microscopic polyangiitis" or "Mixed connective tissue disease" or "Mucha-Habermann disease" or "Pityriasis Lichenoides" or "Multifocal motor neuropathy" or "Multiple sclerosis" or "Myasthenia gravis" or "Myelin oligodendrocyte glycoprotein" or Myositis or Narcolepsy or Neutropenia or "Ocular cicatricial pemphigoid" or "Optic neuritis" or "Palindromic rheumatism" or PANDAS or "Pediatric autoimmune neuropsychiatric disorders" or "Pediatric Autoimmune Neuropsychiatric Disorders Associated with Streptococcal infections" or "Paraneoplastic cerebellar degeneration" or "Paroxysmal nocturnal hemoglobinuria" or "Pars planitis" or “peripheral uveitis” or "Parsonage-Turner syndrome" or "Brachial Plexus Neuritis" or Pemphigus or "Peripheral neuropathy" or "Perivenous encephalomyelitis" or "Pernicious anemia" or "POEMS syndrome" or "Polyarteritis nodosa" or "Polyglandular syndromes" or "Polymyalgia rheumatica" or Polymyositis or "Postmyocardial infarction syndrome" or "Postpericardiotomy syndrome" or "Primary biliary cholangitis" or "Primary sclerosing cholangitis" or "Progesterone dermatitis" or “Autoimmune progesterone dermatitis” or "Progressive hemifacial atrophy" or "Parry Romberg syndrome" or "Facial Hemiatrophy" or Psoriasis or "Psoriatic arthritis" or "Pure red cell aplasia" or "Pyoderma gangrenosum" or Raynaud* or "Reactive arthritis" or "Relapsing polychondritis" or "Restless legs syndrome" or "Retroperitoneal fibrosis" or "Rheumatic fever" or "Rheumatoid arthritis" or Sarcoidosis or "Schmidt syndrome" or "autoimmune polyendocrine syndrome" or Scleritis or Scleroderma or "Sjogren* Disease" or "Stiff person syndrome" or "Susac* syndrome" or "Sympathetic ophthalmia" or "Takayasu* arteritis" OR "Thrombocytopenic purpura" or "Thrombotic thrombocytopenic purpura" or "Thyroid eye disease" OR "Graves Ophthalmopathy" or "Tolosa-Hunt syndrome" or "Transverse myelitis" or "Ulcerative colitis" or "Undifferentiated connective tissue disease" or Uveitis or Vasculitis or Vitiligo or "Vogt-Koyanagi-Harada disease" OR "Uveomeningoencephalitic Syndrome"AND DiGeorge OR “Di George” OR Di-George OR “Pharyngeal Pouch syndrome” OR “Autosomal Dominant Opitz G-Bbb Syndrome” OR “Autosomal Dominant Opitz G Bbb Syndrome” OR “Thymic Aplasia Syndrome” OR Catch22 OR “catch 22” OR “Hypoplasia of Thymus” OR “thymus hypoplasia” OR "parathyroid* hypoplasia" OR “Velocardiofacial Syndrome” OR “Syndrome, Velocardiofacial” OR “VCF Syndrome” OR “Syndrome, VCF” OR “Velo-Cardio-Facial Syndrome” OR “syndrome, Velo-Cardio-Facial” “Velo Cardio Facial Syndrome” OR “Syndrome, Velo-Cardio-Facial” OR “Sedlackova Syndrome” OR “Syndrome, Sedlackova” OR Shprintzen OR 22q11.2DS OR 22q11.2* OR “Conotruncal Anomaly” OR CTAF OR “Takao syndrome” OR “Cayler cardiofacial” OR “Cayler syndrome” OR TBX1 Limited to English |
| **EMBASE 10/5/2023** | autoimmun* OR 'auto immune' OR 'auto immunity' OR autoantibod* OR 'auto antibod*' OR achalasia OR 'addison* disease' OR 'still* disease' OR agammaglobulinemia OR 'alopecia areata' OR amyloidosis OR 'ankylosing spondylitis' OR 'anti-gbm nephritis' OR 'anti tbm nephritis' OR 'anti-glomerular basement membrane disease' OR 'antiphospholipid syndrome' OR 'autoimmune angioedema' OR 'autoimmune dysautonomia' OR 'autoimmune encephalitis' OR 'autoimmune hepatitis' OR 'autoimmune urticaria' OR 'chronic urticaria' OR 'axonal neuropathy' OR 'neuronal neuropathy' OR 'balo disease' OR 'diffuse cerebral sclerosis of schilder' OR 'behcet* disease' OR 'behcet syndrome' OR 'benign mucosal pemphigoid' OR 'bullous pemphigoid' OR 'castleman disease' OR 'celiac disease' OR 'chagas disease' OR 'chronic inflammatory demyelinating polyneuropathy' OR 'chronic recurrent multifocal osteomyelitis' OR 'churg-strauss syndrome' OR 'eosinophilic granulomatosis' OR 'cicatricial pemphigoid' OR 'cogan* syndrome' OR 'cold agglutinin disease' OR 'complex regional pain syndrome*' OR 'sympathetic dystrophy' OR 'congenital heart block' OR 'coxsackie myocarditis' OR 'crest syndrome' OR 'crohn* disease' OR 'dermatitis herpetiformis' OR dermatomyositis OR 'devic* disease' OR 'neuromyelitis optica' OR 'dressler* syndrome' OR 'donath-landsteiner hemolytic anemia' OR endometriosis OR 'eosinophilic esophagitis' OR 'eosinophilic fasciitis' OR 'erythema nodosum' OR 'essential mixed cryoglobulinemia' OR 'evans syndrome' OR fibromyalgia OR 'fibrosing alveolitis' OR 'giant cell arteritis' OR 'temporal arteritis' OR 'giant cell myocarditis' OR glomerulonephritis OR 'goodpasture* syndrome' OR granulomatosis OR 'graves* disease' OR 'guillain barre' OR 'hashimoto* thyroiditis' OR 'hashimoto disease' OR 'hemolytic anemia' OR 'henoch-schonlein purpura' OR 'iga vasculitis' OR 'herpes gestationis' OR 'pemphigoid gestationis' OR 'hidradenitis suppurativa' OR 'acne inversa' OR 'iga nephropathy' OR 'igg 4-related sclerosing disease' OR 'immune thrombocytopenic purpura' OR 'interstitial cystitis' OR 'juvenile arthritis' OR 'juvenile diabetes' OR 'type i diabetes' OR 'diabetes mellitus, type 1' OR 'kawasaki disease' OR 'mucocutaneous lymph node syndrome' OR 'lambert-eaton syndrome' OR 'lichen planus' OR 'lichen sclerosus' OR 'ligneous conjunctivitis' OR 'linear iga disease' OR lupus OR 'lyme disease' OR 'meniere* disease' OR 'microscopic polyangiitis' OR 'mixed connective tissue disease' OR 'mucha-habermann disease' OR 'pityriasis lichenoides' OR 'multifocal motor neuropathy' OR 'multiple sclerosis' OR 'myasthenia gravis' OR 'myelin oligodendrocyte glycoprotein' OR myositis OR narcolepsy OR neutropenia OR 'ocular cicatricial pemphigoid' OR 'optic neuritis' OR 'palindromic rheumatism' OR pandas OR 'pediatric autoimmune neuropsychiatric disorders' OR 'pediatric autoimmune neuropsychiatric disorders associated with streptococcal infections' OR 'paraneoplastic cerebellar degeneration' OR 'paroxysmal nocturnal hemoglobinuria' OR 'pars planitis' OR 'peripheral uveitis' OR 'parsonage-turner syndrome' OR 'brachial plexus neuritis' OR pemphigus OR 'peripheral neuropathy' OR 'perivenous encephalomyelitis' OR 'pernicious anemia' OR 'poems syndrome' OR 'polyarteritis nodosa' OR 'polyglandular syndromes' OR 'polymyalgia rheumatica' OR polymyositis OR 'postmyocardial infarction syndrome' OR 'postpericardiotomy syndrome' OR 'primary biliary cholangitis' OR 'primary sclerosing cholangitis' OR 'progesterone dermatitis' OR 'autoimmune progesterone dermatitis' OR 'progressive hemifacial atrophy' OR 'parry romberg syndrome' OR 'facial hemiatrophy' OR psoriasis OR 'psoriatic arthritis' OR 'pure red cell aplasia' OR 'pyoderma gangrenosum' OR raynaud* OR 'reactive arthritis' OR 'relapsing polychondritis' OR 'restless legs syndrome' OR 'retroperitoneal fibrosis' OR 'rheumatic fever' OR 'rheumatoid arthritis' OR sarcoidosis OR 'schmidt syndrome' OR 'autoimmune polyendocrine syndrome' OR scleritis OR scleroderma OR 'sjogren* disease' OR 'stiff person syndrome' OR 'susac* syndrome' OR 'sympathetic ophthalmia' OR 'takayasu* arteritis' OR 'thrombocytopenic purpura' OR 'thrombotic thrombocytopenic purpura' OR 'thyroid eye disease' OR 'graves ophthalmopathy' OR 'tolosa-hunt syndrome' OR 'transverse myelitis' OR 'ulcerative colitis' OR 'undifferentiated connective tissue disease' OR uveitis OR vasculitis OR vitiligo OR 'vogt-koyanagi-harada disease' OR 'uveomeningoencephalitic syndrome'  AND  digeorge OR 'di george' OR 'pharyngeal pouch syndrome' OR 'autosomal dominant opitz g-bbb syndrome' OR 'autosomal dominant opitz g bbb syndrome' OR 'thymic aplasia syndrome' OR catch22 OR 'catch 22' OR 'hypoplasia of thymus' OR 'thymus hypoplasia' OR 'thymus hypoplasia' OR 'parathyroid* hypoplasia' OR 'velocardiofacial syndrome' OR 'velocardiofacial syndrome' OR 'syndrome, velocardiofacial' OR 'vcf syndrome' OR 'syndrome, vcf' OR 'velo-cardio-facial syndrome' OR 'syndrome, velo-cardio-facial') AND 'velo cardio facial syndrome' OR 'syndrome, velo-cardio-facial' OR 'sedlackova syndrome' OR 'syndrome, sedlackova' OR shprintzen OR 22q11.2ds OR 22q11.2* OR 'conotruncal anomaly' OR ctaf OR 'takao syndrome' OR 'cayler cardiofacial' OR 'cayler syndrome' OR tbx1  #1 AND #2 AND [english]/lim |
| **CINAHL 10/5/2023** | digeorge OR 'di george' OR 'pharyngeal pouch syndrome' OR 'autosomal dominant opitz g-bbb syndrome' OR 'autosomal dominant opitz g bbb syndrome' OR 'thymic aplasia syndrome' OR catch22 OR 'catch 22' OR 'hypoplasia of thymus' OR 'thymus hypoplasia' OR 'thymus hypoplasia' OR 'parathyroid* hypoplasia' OR 'velocardiofacial syndrome' OR 'velocardiofacial syndrome' OR 'syndrome, velocardiofacial' OR 'vcf syndrome' OR 'syndrome, vcf' OR 'velo-cardio-facial syndrome' OR 'syndrome, velo-cardio-facial' AND 'velo cardio facial syndrome' OR 'syndrome, velo-cardio-facial' OR 'sedlackova syndrome' OR 'syndrome, sedlackova' OR shprintzen OR 22q11.2ds OR 22q11.2* OR 'conotruncal anomaly' OR ctaf OR 'takao syndrome' OR 'cayler cardiofacial' OR 'cayler syndrome' OR tbx1  AND  autoimmun* OR 'auto immune' OR 'auto immunity' OR autoantibod* OR 'auto antibod*' OR achalasia OR 'addison* disease' OR 'still* disease' OR agammaglobulinemia OR 'alopecia areata' OR amyloidosis AND 'ankylosing spondylitis' OR 'anti-gbm nephritis' OR 'anti tbm nephritis' OR 'anti-glomerular basement membrane disease' OR 'antiphospholipid syndrome' OR 'autoimmune angioedema' OR 'autoimmune dysautonomia' OR 'autoimmune encephalitis' OR 'autoimmune hepatitis' OR 'autoimmune urticaria' OR 'chronic urticaria' OR 'axonal neuropathy' OR 'neuronal neuropathy' OR 'balo disease' OR 'diffuse cerebral sclerosis of schilder' OR 'behcet* disease' OR 'behcet syndrome' OR 'benign mucosal pemphigoid' OR 'bullous pemphigoid' OR 'castleman disease' OR 'celiac disease' OR 'chagas disease' OR 'chronic inflammatory demyelinating polyneuropathy' OR 'chronic recurrent multifocal osteomyelitis' OR 'churg-strauss syndrome' OR 'eosinophilic granulomatosis' OR 'cicatricial pemphigoid' OR 'cogan* syndrome' OR 'cold agglutinin disease' OR 'complex regional pain syndrome*' OR 'sympathetic dystrophy' OR 'congenital heart block' OR 'coxsackie myocarditis' OR 'crest syndrome' OR 'crohn* disease' OR 'dermatitis herpetiformis' OR dermatomyositis OR 'devic* disease' OR 'neuromyelitis optica' OR 'dressler* syndrome' OR 'donath-landsteiner hemolytic anemia' OR endometriosis OR 'eosinophilic esophagitis' OR 'eosinophilic fasciitis' OR 'erythema nodosum' OR 'essential mixed cryoglobulinemia' OR 'evans syndrome' OR fibromyalgia OR 'fibrosing alveolitis' OR 'giant cell arteritis' OR 'temporal arteritis' OR 'giant cell myocarditis' OR glomerulonephritis OR 'goodpasture* syndrome' OR granulomatosis OR 'graves* disease' OR 'guillain barre' OR 'hashimoto* thyroiditis' OR 'hashimoto disease' OR 'hemolytic anemia' OR 'henoch-schonlein purpura' OR 'iga vasculitis' OR 'herpes gestationis' OR 'pemphigoid gestationis' OR 'hidradenitis suppurativa' OR 'acne inversa' OR 'iga nephropathy' OR 'igg4-related sclerosing disease' OR 'immune thrombocytopenic purpura' OR 'interstitial cystitis' OR 'juvenile arthritis' OR 'juvenile diabetes' OR 'type i diabetes' OR 'diabetes mellitus, type 1' OR 'kawasaki disease' OR 'mucocutaneous lymph node syndrome' OR 'lambert-eaton syndrome' OR 'lichen planus' OR 'lichen sclerosus' OR 'ligneous conjunctivitis' OR 'linear iga disease' OR lupus OR 'lyme disease' OR 'meniere* disease' OR 'microscopic polyangiitis' OR 'mixed connective tissue disease' OR 'mucha-habermann disease' OR 'pityriasis lichenoides' OR 'multifocal motor neuropathy' OR 'multiple sclerosis' OR 'myasthenia gravis' OR 'myelin oligodendrocyte glycoprotein' OR myositis OR narcolepsy OR neutropenia OR 'ocular cicatricial pemphigoid' OR 'optic neuritis' OR 'palindromic rheumatism' OR pandas OR 'pediatric autoimmune neuropsychiatric disorders' OR 'pediatric autoimmune neuropsychiatric disorders associated with streptococcal infections' OR 'paraneoplastic cerebellar degeneration' OR 'paroxysmal nocturnal hemoglobinuria' OR 'pars planitis' OR 'peripheral uveitis' OR 'parsonage-turner syndrome' OR 'brachial plexus neuritis' OR pemphigus OR 'peripheral neuropathy' OR 'perivenous encephalomyelitis' OR 'pernicious anemia' OR 'poems syndrome' OR 'polyarteritis nodosa' OR 'polyglandular syndromes' OR 'polymyalgia rheumatica' OR polymyositis OR 'postmyocardial infarction syndrome' OR 'postpericardiotomy syndrome' OR 'primary biliary cholangitis' OR 'primary sclerosing cholangitis' OR 'progesterone dermatitis' OR 'autoimmune progesterone dermatitis' OR 'progressive hemifacial atrophy' OR 'parry romberg syndrome' OR 'facial hemiatrophy' OR psoriasis OR 'psoriatic arthritis' OR 'pure red cell aplasia' OR 'pyoderma gangrenosum' OR raynaud* OR 'reactive arthritis' OR 'relapsing polychondritis' OR 'restless legs syndrome' OR 'retroperitoneal fibrosis' OR 'rheumatic fever' OR 'rheumatoid arthritis' OR sarcoidosis OR 'schmidt syndrome' OR 'autoimmune polyendocrine syndrome' OR scleritis OR scleroderma OR 'sjogren* disease' OR 'stiff person syndrome' OR 'susac* syndrome' OR 'sympathetic ophthalmia' OR 'takayasu* arteritis' OR 'thrombocytopenic purpura' OR 'thrombotic thrombocytopenic purpura' OR 'thyroid eye disease' OR 'graves ophthalmopathy' OR 'tolosa-hunt syndrome' OR 'transverse myelitis' OR 'ulcerative colitis' OR 'undifferentiated connective tissue disease' OR uveitis OR vasculitis OR vitiligo OR 'vogt-koyanagi-harada disease' OR 'uveomeningoencephalitic syndrome'  Limit to English |
| **Cochrane 10/05/2023** | digeorge OR 'di george' OR 'pharyngeal pouch syndrome' OR 'autosomal dominant opitz g-bbb syndrome' OR 'autosomal dominant opitz g bbb syndrome' OR 'thymic aplasia syndrome' OR catch22 OR 'catch 22' OR 'hypoplasia of thymus' OR 'thymus hypoplasia' OR 'thymus hypoplasia' OR 'parathyroid* hypoplasia' OR 'velocardiofacial syndrome' OR 'velocardiofacial syndrome' OR 'syndrome, velocardiofacial' OR 'vcf syndrome' OR 'syndrome, vcf' OR 'velo-cardio-facial syndrome' OR 'syndrome, velo-cardio-facial' AND 'velo cardio facial syndrome' OR 'syndrome, velo-cardio-facial' OR 'sedlackova syndrome' OR 'syndrome, sedlackova' OR shprintzen OR 22q11.2ds OR 22q11.2* OR 'conotruncal anomaly' OR ctaf OR 'takao syndrome' OR 'cayler cardiofacial' OR 'cayler syndrome' OR tbx1  AND  autoimmun* OR 'auto immune' OR 'auto immunity' OR autoantibod* OR 'auto antibod*' OR achalasia OR 'addison* disease' OR 'still* disease' OR agammaglobulinemia OR 'alopecia areata' OR amyloidosis AND 'ankylosing spondylitis' OR 'anti-gbm nephritis' OR 'anti tbm nephritis' OR 'anti-glomerular basement membrane disease' OR 'antiphospholipid syndrome' OR 'autoimmune angioedema' OR 'autoimmune dysautonomia' OR 'autoimmune encephalitis' OR 'autoimmune hepatitis' OR 'autoimmune urticaria' OR 'chronic urticaria' OR 'axonal neuropathy' OR 'neuronal neuropathy' OR 'balo disease' OR 'diffuse cerebral sclerosis of schilder' OR 'behcet* disease' OR 'behcet syndrome' OR 'benign mucosal pemphigoid' OR 'bullous pemphigoid' OR 'castleman disease' OR 'celiac disease' OR 'chagas disease' OR 'chronic inflammatory demyelinating polyneuropathy' OR 'chronic recurrent multifocal osteomyelitis' OR 'churg-strauss syndrome' OR 'eosinophilic granulomatosis' OR 'cicatricial pemphigoid' OR 'cogan* syndrome' OR 'cold agglutinin disease' OR 'complex regional pain syndrome*' OR 'sympathetic dystrophy' OR 'congenital heart block' OR 'coxsackie myocarditis' OR 'crest syndrome' OR 'crohn* disease' OR 'dermatitis herpetiformis' OR dermatomyositis OR 'devic* disease' OR 'neuromyelitis optica' OR 'dressler* syndrome' OR 'donath-landsteiner hemolytic anemia' OR endometriosis OR 'eosinophilic esophagitis' OR 'eosinophilic fasciitis' OR 'erythema nodosum' OR 'essential mixed cryoglobulinemia' OR 'evans syndrome' OR fibromyalgia OR 'fibrosing alveolitis' OR 'giant cell arteritis' OR 'temporal arteritis' OR 'giant cell myocarditis' OR glomerulonephritis OR 'goodpasture* syndrome' OR granulomatosis OR 'graves* disease' OR 'guillain barre' OR 'hashimoto* thyroiditis' OR 'hashimoto disease' OR 'hemolytic anemia' OR 'henoch-schonlein purpura' OR 'iga vasculitis' OR 'herpes gestationis' OR 'pemphigoid gestationis' OR 'hidradenitis suppurativa' OR 'acne inversa' OR 'iga nephropathy' OR 'immune thrombocytopenic purpura' OR 'interstitial cystitis' OR 'juvenile arthritis' OR 'juvenile diabetes' OR 'type i diabetes' OR 'diabetes mellitus, type 1' OR 'kawasaki disease' OR 'mucocutaneous lymph node syndrome' OR 'lambert-eaton syndrome' OR 'lichen planus' OR 'lichen sclerosus' OR 'ligneous conjunctivitis' OR 'linear iga disease' OR lupus OR 'lyme disease' OR 'meniere* disease' OR 'microscopic polyangiitis' OR 'mixed connective tissue disease' OR 'mucha-habermann disease' OR 'pityriasis lichenoides' OR 'multifocal motor neuropathy' OR 'multiple sclerosis' OR 'myasthenia gravis' OR 'myelin oligodendrocyte glycoprotein' OR myositis OR narcolepsy OR neutropenia OR 'ocular cicatricial pemphigoid' OR 'optic neuritis' OR 'palindromic rheumatism' OR pandas OR 'pediatric autoimmune neuropsychiatric disorders' OR 'pediatric autoimmune neuropsychiatric disorders associated with streptococcal infections' OR 'paraneoplastic cerebellar degeneration' OR 'paroxysmal nocturnal hemoglobinuria' OR 'pars planitis' OR 'peripheral uveitis' OR 'parsonage-turner syndrome' OR 'brachial plexus neuritis' OR pemphigus OR 'peripheral neuropathy' OR 'perivenous encephalomyelitis' OR 'pernicious anemia' OR 'poems syndrome' OR 'polyarteritis nodosa' OR 'polyglandular syndromes' OR 'polymyalgia rheumatica' OR polymyositis OR 'postmyocardial infarction syndrome' OR 'postpericardiotomy syndrome' OR 'primary biliary cholangitis' OR 'primary sclerosing cholangitis' OR 'progesterone dermatitis' OR 'autoimmune progesterone dermatitis' OR 'progressive hemifacial atrophy' OR 'parry romberg syndrome' OR 'facial hemiatrophy' OR psoriasis OR 'psoriatic arthritis' OR 'pure red cell aplasia' OR 'pyoderma gangrenosum' OR raynaud* OR 'reactive arthritis' OR 'relapsing polychondritis' OR 'restless legs syndrome' OR 'retroperitoneal fibrosis' OR 'rheumatic fever' OR 'rheumatoid arthritis' OR sarcoidosis OR 'schmidt syndrome' OR 'autoimmune polyendocrine syndrome' OR scleritis OR scleroderma OR 'sjogren* disease' OR 'stiff person syndrome' OR 'susac* syndrome' OR 'sympathetic ophthalmia' OR 'takayasu* arteritis' OR 'thrombocytopenic purpura' OR 'thrombotic thrombocytopenic purpura' OR 'thyroid eye disease' OR 'graves ophthalmopathy' OR 'tolosa-hunt syndrome' OR 'transverse myelitis' OR 'ulcerative colitis' OR 'undifferentiated connective tissue disease' OR uveitis OR vasculitis OR vitiligo OR 'vogt-koyanagi-harada disease' OR 'uveomeningoencephalitic syndrome'  limited to English |
