# Supplementary material for: A Systematic Review of Autoimmunity in 22q11.2 Deletion Syndrome
Source: Expert Rev Mol Med. 2026 Jan 8;28:e12. doi: 10.1017/erm.2026.10031 (PMC13148428; doi:10.1017/erm.2026.10031)
Supplement: Ogunsola et al. supplementary material 2 — Ogunsola et al. supplementary material [file S1462399426100313sup002.docx]

| Molecular, cellular and physiological system functions |  | Genes (or gene products) in the 22q11 TDR implicated | | | | |
| --- | --- | --- | --- | --- | --- | --- |
| DNA Replication, Recombination, and Repair |  | AIFM3,CDC45,DGCR8,HIRA,KLHL22,mir-1286,mir-1306,TBX1 | | | | |
| Nervous System Development and Function |  | CLDN5,COMT,CRKL,DGCR8,GNB1L,HIRA,PRODH,RTN4R,SEPTIN5,SNAP29,TBX1,ZDHHC8 | | | | |
| Cardiovascular System Development and Function |  | CLDN5,COMT,CRKL,DGCR8,HIRA,RTN4R,SERPIND1,TBX1 | | | | |
| Embryonic Development |  | AIFM3,CLDN5,COMT,CRKL,DGCR8,HIRA,PI4KA,RANBP1,RIMBP3,SERPIND1,TBX1,TSSK2 | | | | |
| Organismal Development |  | CLDN5,COMT,CRKL,DGCR8,GP1BB,HIRA,PI4KA,RANBP1,RIMBP3,SERPIND1,TBX1,TSSK2 | | | | |
| Tissue Development |  | CLDN5,COMT,CRKL,DGCR8,HIRA,PI4KA,RANBP1,RIMBP3,RTN4R,SERPIND1,TBX1,TSSK2 | | | | |
| Endocrine System Development and Function |  | COMT,CRKL,PI4KA,RANBP1,RIMBP3,TBX1,TSSK2 | | | | |
| Organ Development |  | CLDN5,COMT,CRKL,DGCR8,HIRA,PI4KA,RANBP1,RIMBP3,RTN4R,TBX1,TSSK2 | | | | |
| Cell Signaling |  | CRKL,LZTR1,RTN4R,TBX1,TSSK2 | | | |  |
| Cellular Assembly and Organization |  | AIFM3,ARVCF,CRKL,DGCR8,KLHL22,RANBP1,RIMBP3,RTN4R,SCARF2,SEPTIN5,SNAP29 | | | | |
| Behavior |  | CLDN5,COMT,DGCR2,GNB1L,RTN4R,SEPTIN5,TBX1,ZDHHC8 | | | | |
| Cell Death and Survival |  | AIFM3,ARVCF,COMT,CRKL,DGCR8,PRODH,RANBP1,RTN4R,SLC25A1,SNAP29,TBX1,UBE2L3 | | | | |
| Amino Acid Metabolism |  | COMT,PRODH | |  |  |  |
| Cell-To-Cell Signaling and Interaction |  | ARVCF,COMT,CRKL,LZTR1,PRODH,RIMBP3,RTN4R,SCARF2,SEPTIN5,SERPIND1,SNAP29,UBE2L3,ZDHHC8 | | | | |
| Cellular Development |  | CRKL,DGCR8,PRODH,RANBP1,RIMBP3,RTN4R,SERPIND1,TBX1,TSSK2 | | | | |
| Cellular Growth and Proliferation |  | CRKL,DGCR8,PI4KA,PRODH,RANBP1,RIMBP3,RTN4R,SERPIND1,TBX1,TSSK2 | | | | |
| Cellular Movement |  | CRKL,DGCR8,SEPTIN5,SERPIND1,SNAP29 | | | | |
| Connective Tissue Development and Function |  | CLDN5,COMT,CRKL,DGCR8,GP1BB,RTN4R,SCARF2,TBX1 | | | | |
| Drug Metabolism |  | COMT,SEPTIN5,SNAP29 | | |  |  |
| Energy Production |  | PRODH |  |  |  |  |
| Lipid Metabolism |  | COMT,PI4KA,SLC25A1,ZDHHC8 | | | |  |
| Molecular Transport |  | COMT,PI4KA,RTN4R,SEPTIN5,SLC25A1,SNAP29,ZDHHC8 | | | | |
| Organ Morphology |  | COMT,DGCR8,PI4KA,TBX1 | | |  |  |
| Post-Translational Modification |  | COMT,CRKL,PI4KA,PRODH,TBX1,TSSK2,ZDHHC8 | | | | |
| Skeletal and Muscular System Development and Function |  | COMT,CRKL,DGCR8,SERPIND1,TBX1 | | | |  |
| Small Molecule Biochemistry |  | COMT,PI4KA,PRODH,SEPTIN5,SLC25A1,SNAP29,ZDHHC8 | | | | |
| Tissue Morphology |  | DGCR8,GP1BB,HIRA,PI4KA,RTN4R,SERPIND1,TBX1 | | | | |
| Organismal Survival |  | CDC45,CRKL,DGCR8,HIRA,PI4KA,SERPIND1,UBE2L3 | | | | |
| Reproductive System Development and Function |  | COMT,CRKL,HIRA,PI4KA,RANBP1,RIMBP3,SLC25A1,TSSK2 | | | | |
| Cellular Function and Maintenance |  | CRKL,DGCR8,LZTR1,RANBP1,RTN4R,SEPTIN5,SERPIND1,SNAP29,TBX1,UBE2L3 | | | | |
| Cell Morphology |  | CRKL,GP1BB,PRODH,RANBP1,RIMBP3,RTL10,RTN4R,SERPIND1,SNAP29 | | | | |
| Cellular Compromise |  | CLDN5,COMT,CRKL,TBX1 | | |  |  |
| Vitamin and Mineral Metabolism |  | COMT |  |  |  |  |
| Cell Cycle |  | CDC45,CRKL,KLHL22,PI4KA,PRODH,RANBP1,SLC25A1 | | | | |
| Digestive System Development and Function |  | CLDN5,TBX1 | |  |  |  |
| Renal and Urological System Development and Function |  | RANBP1,RTN4R | |  |  |  |
| Carbohydrate Metabolism |  | PI4KA |  |  |  |  |
| Cell-mediated Immune Response |  | DGCR8 |  |  |  |  |
| Lymphoid Tissue Structure and Development |  | DGCR8,TBX1 | |  |  |  |
| Auditory and Vestibular System Development and Function |  | TBX1 |  |  |  |  |
| Hematological System Development and Function |  | CRKL,DGCR8,GP1BB,LZTR1,SERPIND1,UBE2L3 | | | | |
| Nucleic Acid Metabolism |  | COMT |  |  |  |  |
| Organismal Functions |  | COMT,TBX1 | |  |  |  |
| Humoral Immune Response |  | DGCR8 |  |  |  |  |
| Visual System Development and Function |  | DGCR8 |  |  |  |  |
| Cellular Response to Therapeutics |  | CLDN5 |  |  |  |  |
| Hematopoiesis |  | DGCR8 |  |  |  |  |
| Immune Cell Trafficking |  | CRKL |  |  |  |  |
| Respiratory System Development and Function |  | TBX1 |  |  |  |  |

Note: We uploaded all genes within the 22q11 typically-deleted region (including micro-RNAs) into Qiagen Ingenuity Pathway Analysis (IPA), which draws on a scientist curated knowledgebase (https://digitalinsights.qiagen.com/products-overview/qiagen-knowledge-base/ ). Genes were mapped to relevant biological functions. The IPA functional annotations integrate GO data (www.geneontology.org), but the IPA knowledgebase is more extensive and complete.
